# NIR‐II Light‐Triggered Electron Flow Initiates Cuproptosis‐Centered Thermoelectric‐Immunotherapy for Breast Cancer

**DOI:** 10.1002/advs.76368

**Published:** 2026-06-30

**Authors:** Boyu Yuan, Qin Fan, Shushu Chu, Shining Yang, Yuechao Yang, Chenxi Zhang, Jinqiao Zhang, Xinran Qu, Yiju Wei, Xin Wang, Ziliang Dong

**Affiliations:** ^1^ Department of Clinical Pharmacy, Shandong Key Laboratory of Digital Diagnosis and Treatment of Thoracic Oncology The First Affiliated Hospital of Shandong First Medical University & Shandong Provincial Qianfoshan Hospital Jinan Shandong P. R. China; ^2^ Science and Technology Innovation Center Shandong First Medical University & Shandong Academy of Medicine Sciences Jinan Shandong P. R. China; ^3^ State Key Laboratory of Flexible Electronics (LoFE) & Institute of Advanced Materials (IAM) Nanjing University of Posts & Telecommunications Nanjing Jiangsu P. R. China; ^4^ Faculty of Light Industry, State Key Laboratory of Green Papermaking and Resource Recycling Qilu University of Technology (Shandong Academy of Sciences) Jinan P. R. China; ^5^ School of Life Science Shandong First Medical University & Shandong Academy of Medica science Tai'an Shandong China

**Keywords:** copper valence conversion, cuproptosis, electron flow, thermoelectric nanoreactor, tumor therapy

## Abstract

Cuproptosis, a copper‐dependent form of regulated cell death, holds significant promise for oncology, but its therapeutic utility is constrained by the inefficient intratumoral generation of the bioactive Cu^+^ species. To this end, we report a NIR‐II light‐activatable thermoelectric nanoreactor (Te@PDA‐Cu^II^) designed to achieve spatiotemporally controlled Cu^+^ production for enhanced cancer therapy. The nanoreactor comprises a tellurium nanorod core, which exhibits a strong thermoelectric effect under 1064 nm light irradiation, coated with a polydopamine (PDA) layer that serves both as a Cu^2+^ chelation scaffold and an electron‐conducting interface. Photothermal activation generates a directional electron flow from the Te core, which is efficiently relayed through the PDA layer to reduce surface‐bound Cu^2+^ to Cu^+^. Such in situ valence conversion potently triggers cuproptosis via dihydrolipoamide S‐acetyltransferase (DLAT) aggregation and Fe─S cluster destabilization. The ensuing cuproptosis initiates immunogenic cell death (ICD), promoting dendritic cell maturation and cytotoxic T lymphocyte infiltration. In orthotopic and metastatic breast cancer models, this thermoelectric‐immunological cascade not only eradicates primary tumors but also synergizes with aPD‐1 checkpoint blockade to suppress distant metastases and establish durable immune memory. This work establishes a physical energy‐driven strategy for precise cuproptosis activation and demonstrates its potential to amplify cancer immunotherapy.

## Graphical abstract

A NIR‐II light‐activatable thermoelectric nanoreactor converts surface‐bound Cu^2+^ into bioactive Cu^+^ via directed electron flow. This spatiotemporally controlled reduction triggers potent cuproptosis and immunogenic cell death, eradicating primary tumors and, together with aPD‐1 therapy, suppressing metastases while establishing durable immune memory.

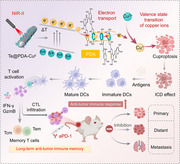

## 1 Introduction

Cuproptosis has emerged as a distinct, copper‐dependent form of regulated cell death, presenting a novel therapeutic avenue in cancer [1, 2, 3]. Unlike apoptosis or ferroptosis, cuproptosis is triggered specifically by intracellular copper(I) (Cu^+^) ions, which directly target lipoylated proteins in the tricarboxylic acid (TCA) cycle, such as DLAT [4, 5]. Such interaction induces fatal protein aggregation and subsequent metabolic collapse. The efficacy of this mechanism, however, is inherently contingent upon the localized generation of the bioactive Cu^+^ valence [6, 7]. Current strategies primarily rely on the administration of copper ions or ionophores, which suffer from critical limitations: systemic toxicity, poor tumor‐specific bioavailability, and, most importantly, the inability to ensure the precise and sufficient in situ conversion of the commonly available Cu^2+^ into the toxic Cu^+^ species. Thus, achieving precise in situ Cu^+^ generation remains a pivotal yet unresolved obstacle, limiting the practical application of cuproptosis in cancer.

Thermoelectric materials, capable of converting thermal energy directly into electricity through the Seebeck effect, offer a unique platform for spatiotemporally controlled biochemical modulation [8, 9, 10]. Upon exposure to a temperature gradient, these materials generate an internal electric field that drives the directional flow of charge carriers (electrons and holes) [11]. This intrinsic property has been leveraged in biomedical applications, such as thermoelectric catalytic therapy, where mechanical or thermal energy is harnessed to produce reactive oxygen species [12, 13, 14]. Beyond catalysis, the generated “hot electrons” represent a potent, physically derived reducing force. If efficiently harvested and delivered, these electrons could serve as a precise, external energy‐controlled tool to drive specific redox reactions in situ, such as the reduction of metal ions within a biological milieu, opening new avenues for non‐invasive bio‐intervention.

**SCHEME 1:**
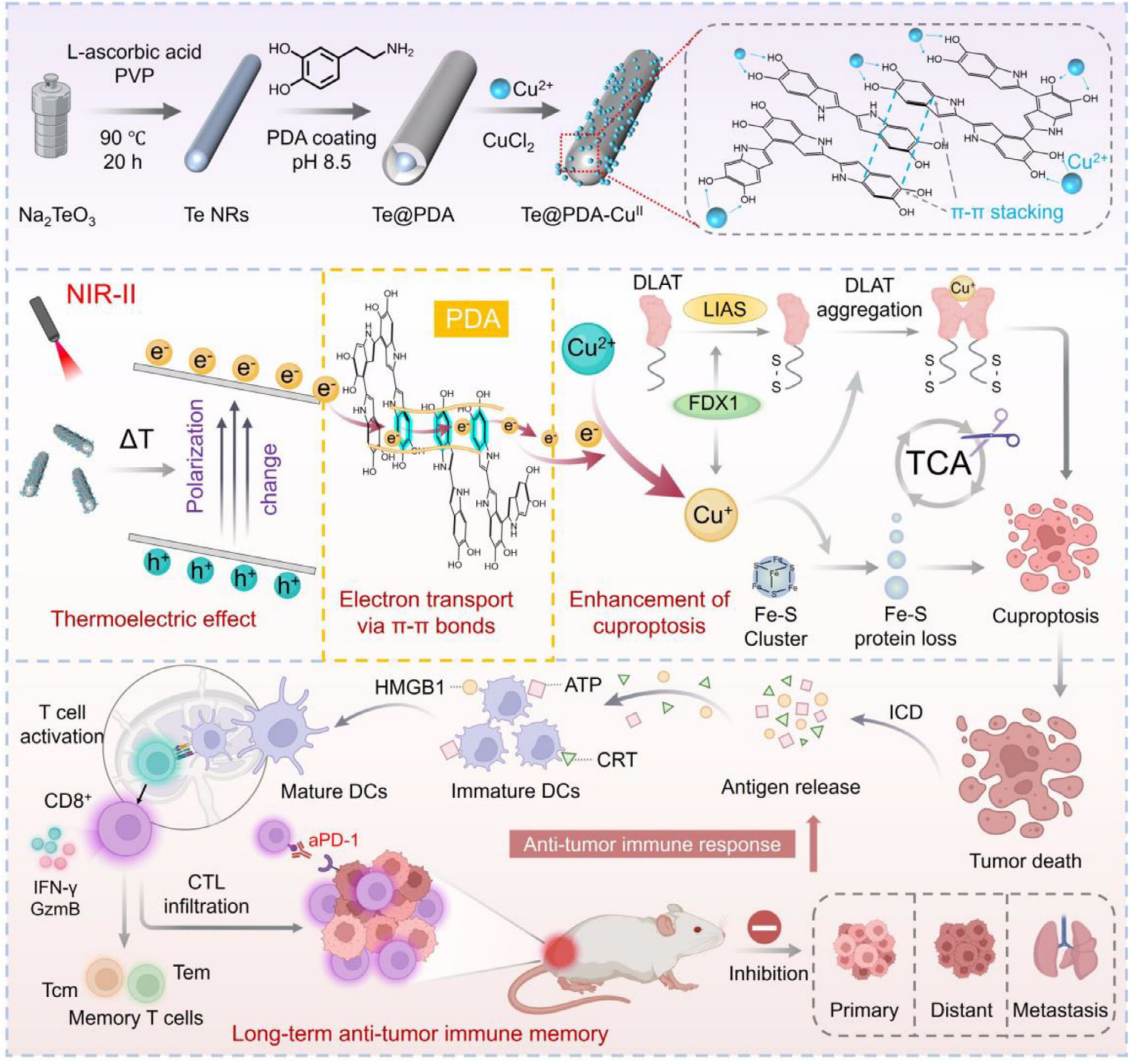
Schematic illustration of NIR‐II‐activated Te@PDA‐Cu^II^ nanoreactor for cuproptosis‐centered thermoelectric‐immunological therapy. (A) Te NRs are coated with a PDA layer, which enables efficient Cu^2+^ chelation to form the Te@PDA‐Cu^II^ nanoreactor. (B) Upon NIR‐II laser irradiation, the thermoelectric effect of Te NRs generates directional electron flow. The conductive PDA interlayer relays these electrons to reduce Cu^2+^ to Cu^+^, igniting cuproptosis through DLAT aggregation and Fe─S cluster destabilization and inducing ICD, thus promoting DC maturation and CTL infiltration. (C) In orthotopic breast cancer models, such thermoelectric‐immunological cascade effectively ablates primary tumors. When combined with aPD‐1 checkpoint blockade, it suppresses distant metastases and establishes long‐term immune memory, providing a physical energy‐driven strategy for cuproptosis‐enhanced immunotherapy.

Herein, we introduce a NIR‐II laser‐activated thermoelectric nanoreactor designed to harness this physical energy for precise Cu^+^ generation. Our construct, Te@PDA‐Cu^II^, is built on a tellurium nanorod (Te NR) core, chosen for its high thermoelectric coefficient, and coated with a PDA layer. Such a PDA shell serves a dual purpose: it acts as a scaffold for the stable chelation of Cu^2+^ ions and, crucially, functions as an electron‐conducting relay. Upon 1064 nm laser irradiation, the photothermal effect creates a steep temperature gradient across the Te core, triggering a robust thermoelectric current. The resulting electron flow is directionally channeled through the conductive PDA interlayer, reducing the surface‐bound Cu^2+^ to Cu^+^ with high spatiotemporal precision. This localized amplification of the Cu^+^ pool ignites a potent cuproptotic cascade, which in turn triggers ICD. The release of damage‐associated molecular patterns (DAMPs) subsequently remodels the immunosuppressive tumor microenvironment, initiating a systemic thermoelectric‐immunological cascade. In orthotopic and metastatic 4T1 breast cancer models, this integrated nanoreactor not only ablates primary tumors but also synergizes powerfully with aPD‐1 immune checkpoint blockade to suppress distant metastases and establish long‐term immune memory (Scheme 1). Our work introduces a paradigm for translating physical energy into a precise biochemical signal, offering a novel and effective strategy for cuproptosis‐enhanced cancer immunotherapy.

## 2 Results

### 2.1 Construction and Characterization of Te@PDA‐Cu^II^ Thermoelectric Nanoreactors

**FIGURE 1:**
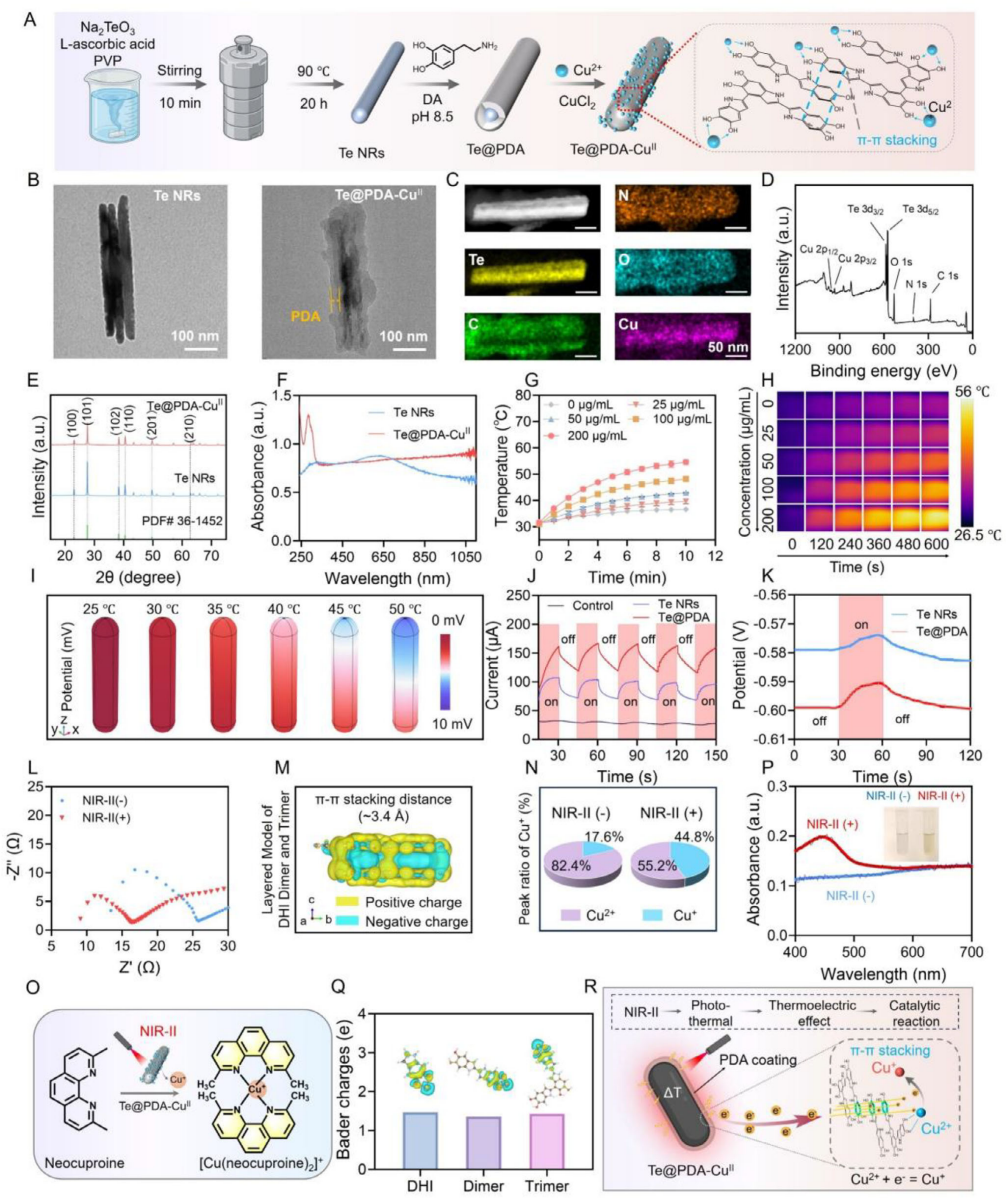
Construction and characterization of Te@PDA‐Cu^II^ thermoelectric nanoreactors (A) Schematic representation of the Te@PDA‐Cu^II^ preparation process. (B, C) TEM images of bare Te NRs, Te@PDA‐Cu^II,^ and corresponding elemental mapping images of Te@PDA‐Cu^II^. (D) Typical XPS spectrum of Te@PDA‐Cu^II^. (E) XRD patterns of Te NRs and Te@PDA‐Cu^II^. (F) UV–vis‐NIR absorbance spectra of Te NRs and Te@PDA‐Cu^II^. (G, H) Temperature rise curves (G) and infrared thermal images (H) of Te@PDA‐Cu^II^ at different concentrations with NIR‐II laser irradiation (0.6 W cm^−2^, 10 min). (I) Finite element simulation of the thermoelectric potential distribution on Te@PDA surfaces at different temperatures. (J, K) Thermoelectric current (J) and potential (K) of Te NRs and Te@PDA under NIR‐II laser irradiation. (L) Electrochemical impedance spectroscopy of Te@PDA with or without NIR‐II laser irradiation. (M) Charge density difference of the stacked DHI dimer and trimer models (yellow and cyan correspond to positive and negative differences, respectively). (N) Relative proportion changes of Cu^+^ and Cu^2+^ species in Te@PDA‐Cu^II^ before and after NIR‐II laser irradiation. (O, P) The reaction mechanism of Cu^+^ detection by neocuproine (O) and the corresponding UV–vis absorbance spectra of neocuproine treated with Te@PDA‐Cu^II^ and NIR‐II stimulation (P). (Q) Charge density difference maps of copper coordinated with DHI monomer, dimer, and trimer (yellow and cyan correspond to positive and negative differences, respectively), along with the Bader charge analysis of the corresponding copper ions. (R) Schematic illustration of the NIR‐II‐activated thermoelectric cascade catalysis mechanism of Te@PDA‐Cu^II^ for Cu^2+^ reduction to Cu^+^.

The synthesis of Te@PDA‐Cu^II^ nanoreactors is schematically illustrated in Figure 1A. Te NRs were first synthesized via a modified hydrothermal method [15, 16]. Subsequently, a polydopamine shell was uniformly coated onto the Te NRs through in situ polymerization of dopamine under weak alkaline conditions, yielding Te@PDA [17]. Finally, Cu^2+^ was chelated onto the surface via the abundant catechol groups of the PDA layer, resulting in the final product, Te@PDA‐Cu^II^. Transmission electron microscopy (TEM) images revealed that the hydrothermally synthesized Te NRs were several hundred nanometers in length. A uniform PDA coating with a thickness of ∼10 nm was observed on the surface (Figure 1B). Energy‐dispersive X‐ray spectroscopy (EDS) elemental mapping confirmed the homogeneous distribution of Te, C, N, O, and Cu throughout the structure (Figure 1C). X‐ray photoelectron spectroscopy (XPS) survey spectra further verified the presence of these elements (Figure 1D). Zeta potential measurements showed a slight negative shift after PDA coating, followed by a significant positive recovery upon Cu^2+^ chelation, preliminarily confirming the successful surface modifications (Figure S1). X‐ray diffraction (XRD) analysis indicated that the diffraction peaks of Te@PDA‐Cu^II^ matched well with the standard pattern for tellurium (PDF#36‐1452) (Figure 1E). Fourier‐transform infrared (FTIR) spectroscopy exhibited characteristic PDA absorption bands at ∼3380 cm^−1^ (attributed to O─H stretch), ∼1600 cm^−1^ (aromatic C═C), and ∼1266 cm^−1^ (C─O stretch) (Figure S2).

Subsequently, the thermoelectric capability of the Te@PDA‐Cu^II^ was investigated. Ultraviolet‐visible‐near‐infrared (UV–vis‐NIR) spectroscopy revealed a significantly enhanced absorption intensity for Te@PDA‐Cu^II^ in the NIR‐II region, particularly around 1064 nm, after PDA coating and copper ion coordination, laying the foundation for efficient photothermal conversion (Figure 1F). Then, it was found that the Te@PDA‐Cu^II^ exhibited excellent photothermal performance under 1064 nm NIR‐II laser irradiation. Monitoring with an infrared thermal imaging camera demonstrated a noticeable temperature increase, which showed clear dependence on both Te@PDA‐Cu^II^ concentration and laser power density, confirming its highly efficient and controllable photothermal conversion capability (Figure 1G,H and Figure S3). Moreover, Te@PDA‐Cu^II^ exhibited excellent thermal stability, maintaining consistent and reproducible heating performance across multiple laser on/off cycles without significant attenuation (Figure S4).

Polydopamine, a widely recognized electron‐transfer medium, was thus incorporated not only to enhance photothermal conversion but also to serve as an efficient electron mediator [18, 19]. Inspired by the thermoelectric nature of tellurium nanorods, we hypothesized that the NIR‑II‑laser induced temperature gradient would generate a thermoelectric current, and further postulated that the conductive PDA layer would facilitate hot‑electron extraction and transfer, thereby amplifying the overall thermoelectric response.

First, solid‐state UV–vis spectroscopy revealed that the introduction of PDA lowered the bandgap of Te NRs, decreasing from ∼3.01 to ∼2.23 eV, which indicates facilitated electron transition (Figure S5). Meanwhile, via finite element simulation analysis, it was shown that the thermoelectric potential of Te@PDA exhibited a marked temperature‐dependent increase, enabling a built‐in electric field that drives hot electron transport (Figure 1I). The photoresponsive thermoelectric behavior was then investigated by measuring the thermal current and potential of Te NRs and Te@PDA under NIR‐II laser irradiation using an electrochemical workstation. As shown in Figure 1J,K, while no pyroelectric current was detected in the control group, Te NRs exhibited discernible photoresponsive current and potential signals under alternating NIR‐II laser irradiation. Notably, the Te@PDA group demonstrated a significantly stronger thermoelectric response compared to pure Te NRs, providing direct evidence that the PDA layer functions as an effective electron extractor, thereby greatly promoting the extraction and separation of hot carriers. Electrochemical impedance spectroscopy (EIS) further revealed that Te@PDA displayed a smaller Nyquist semicircle radius compared to the non‑irradiated group after 5 min of laser irradiation, indicating that the NIR‑II induced heating generation effectively reduced the interfacial charge‑transfer resistance and thus accelerated electron transport (Figure 1L).

Then, to further elucidate the underlying conductive mechanism of PDA, atomic‐level theoretical calculations were performed. First, Electron Localization Function (ELF) analysis revealed that the electron pairs of 5,6‐dihydroxyindole (DHI) oligomers (from monomer to trimer) were strictly localized along the C─C and C─N covalent bonds, with hollow aromatic ring centers, confirming the sp^2^‐hybridized covalent framework in PDA that enables intermolecular π‐π stacking (Figure S6). Building on this structural basis, charge density difference (CDD) analysis of stacked layered models (dimer and trimer complexes) showed significant interlayer charge redistribution, evidencing strong electronic coupling and efficient interlayer charge transfer (Figure 1M). Thus, the intramolecular sp^2^‐hybridized skeleton and the intermolecular π‐π stacking cooperatively construct a highly efficient electron‐transport channel for PDA.

Next, to verify whether the generated hot electrons could reduce Cu^2+^ to Cu^+^, XPS spectroscopy was used to analyze the Cu^+^/Cu^2+^ ratio in Te@PDA‐Cu^II^ before and after NIR‐II laser irradiation. It was found that the proportion of Cu^+^ in Te@PDA‐Cu^II^ increased markedly from 17.6% to 44.8% after NIR‐II laser exposure (Figure 1N and Figure S7). We also utilized Neocuproine, a specific indicator for Cu^+^, which forms a yellow complex [Cu(neocuproine)_2_]^+^ to detect the generation of Cu^+^ (Figure 1O) [20]. UV‑Vis absorption spectrum of Te@PDA‐Cu^II^ after NIR‐II laser irradiation exhibited a characteristic peak at 452 nm corresponding to the [Cu(neocuproine)_2_]^+^ complex, whereas no significant signal was detected in the non‑irradiated group (Figure 1P). Moreover, Density Functional Theory (DFT) calculations were also performed. Coordination models of Cu^2+^ chelated with different polymerization states of PDA, including DHI monomer, dimer, and trimer, were constructed. Charge density difference (CDD) analysis revealed pronounced electron redistribution upon chelation, with substantial accumulation localized around the copper ion and corresponding depletion on the coordinating heteroatoms and DHI backbone, confirming a strong ligand‐to‐metal charge transfer (LMCT) pathway from PDA to the copper center. Furthermore, Bader charge analysis further quantified significant electron acquisition by copper across all complexes, fundamentally driving the reduction of Cu^2+^ to Cu^+^ (Figure 1Q). Therefore, those results demonstrate that thermoelectrically generated electrons are delivered through the PDA interlayer to reduce Cu^2+^ to Cu^+^ in situ, which provides the chemical impetus for triggering subsequent cuproptosis (Figure 1R).

### 2.2 Te@PDA‐Cu^II^ Thermoelectric Nanoreactors Trigger Cuproptosis in Vitro

**FIGURE 2:**
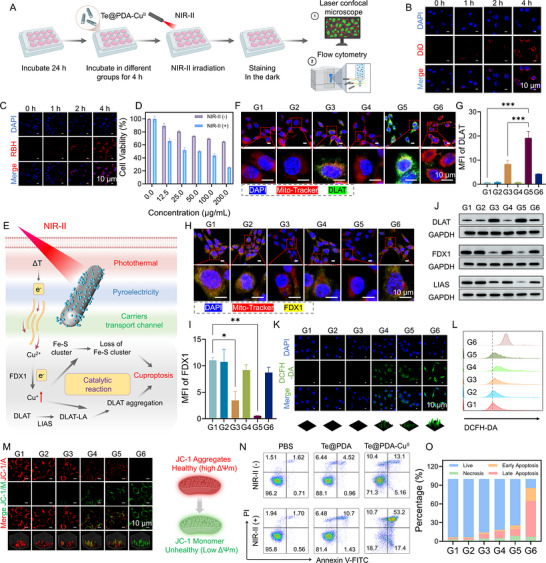
Te@PDA‐Cu^II^ thermoelectric nanoreactors trigger cuproptosis in vitro. (A) Schematic illustration of the cellular treatment procedure. (B) Confocal fluorescence imaging showing the time‐dependent cellular uptake of DiD‐labeled Te@PDA‐Cu^II^ in 4T1 cells. (C) Intracellular accumulation of copper ion monitored by CLSM. (D) Relative viability of 4T1 cells treated with various concentrations of Te@PDA‐Cu^II^ with or without NIR‐II laser irradiation. (E) Schematic illustration of the mechanism underlying Te@PDA‐Cu^II^‐triggered cuproptosis under NIR‐II laser irradiation. (F, G) Immunofluorescence images (F) and corresponding quantitative mean fluorescence intensity (MFI) (G) of DLAT in 4T1 cells following different treatments. (H, I) Immunofluorescence images (H) and corresponding MFI of FDX1 (I) following different treatments. (J) Western blot analysis of DLAT, FDX1, and LIAS expression levels under different treatments. G1–G6 in Figure F–J are noted as follows: G1: PBS, G2: Te@PDA, G3: Te@PDA‐Cu^II^, G4: Te@PDA‐Cu^II^ + TTM, G5: Te@PDA‐Cu^II^ + NIR‐II, G6: Te@PDA‐Cu^II^ + NIR‐II + TTM. (K, L) Confocal fluorescence imaging of intracellular ROS generation (K) and corresponding flow cytometric analysis (L). (M) Confocal fluorescence imaging showing mitochondrial membrane potential changes by JC‐1 staining in 4T1 cells under different treatments. (N, O) Flow cytometric analysis of cell apoptosis (N) and corresponding quantitative apoptosis rates (O) under different treatments. G1–G6 in Figure K–O are noted as follows: G1: PBS, G2: NIR‐II, G3: Te@PDA, G4: Te@PDA + NIR‐II, G5: Te@PDA‐Cu^II^, G6: Te@PDA‐Cu^II^ + NIR‐II. Data are presented as mean ± SEM. The p‐values were determined using one‐way ANOVA followed by Tukey's post hoc test for multiple comparisons; ^*^ *p* < 0.05, ^**^ *p* < 0.01, ^***^ *p* < 0.001.

Encouraged by the exceptional thermoelectric capability of Te@PDA‐Cu^II^ nanoreactors to convert Cu^2+^ to Cu^+^, we systematically evaluated their in vitro capacity to induce cuproptosis (Figure 2A). We first investigated the endocytosis of Te@PDA‐Cu^II^ by 4T1 breast cancer cells. As shown in Figure 2B, confocal laser scanning microscopy (CLSM) images revealed time‐dependent enhancement of fluorescence signals from DiD‐labeled Te@PDA‐Cu^II^ within the cells. Subsequently, the intracellular accumulation of copper ions was confirmed using the copper‐specific fluorescent probe Rhodamine B hydrazide (RBH) by both CLSM imaging and flow cytometry (Figure 2C and Figure S8). Subsequently, the therapeutic efficacy of Te@PDA‐Cu^II^ nanoreactors against 4T1 cells with NIR‐II laser irradiation was evaluated via 3‐(4,5‐Dimethylthiazol‐2‐yl)‐2,5‐diphenyltetrazolium bromide (MTT) assay. As displayed in Figure 2D, Te@PDA‑Cu^II^ alone induced a concentration‑dependent reduction in 4T1 cell viability. Notably, co‑treatment with NIR‑II laser irradiation dramatically enhanced its cytotoxicity. At an equivalent concentration of 200 µg mL^−1^, the cell‐killing efficacy of Te@PDA‑Cu^II^ + NIR‑II irradiation reached 70.1%, which was significantly higher than that of Te@PDA‑Cu^II^ alone (47.7%) and Te@PDA + NIR‑II irradiation (52.2%), respectively (Figure S9). Furthermore, to evaluate the in vitro biosafety of the nanoreactors, cytotoxicity assays were conducted on the non‐cancerous NIH‐3T3 murine fibroblast cell line. As shown in Figure S10, the Te@PDA‐Cu^II^ nanoreactors exhibited low cytotoxicity even under NIR‐II irradiation, possibly due to the reduced sensitivity of normal cells to cuproptosis‐related metabolic stress.

To elucidate the mechanism underlying the cytotoxicity induced by Te@PDA‐Cu^II^ + NIR‐II irradiation, we investigated the pathways associated with cuproptosis. Excessive intracellular copper accumulation triggers the toxic aggregation of lipoylated DLAT and destabilizes Fe─S cluster proteins such as ferredoxin‐1 (FDX1) and lipoyl synthase (LIAS), ultimately impairing the TCA cycle (Figure 2E). Using CLSM, we visualized these hallmarks in 4T1 cells. While only minimal DLAT oligomerization signals were observed in PBS‐ or Te@PDA‐treated groups, Te@PDA‐Cu^II^ alone induced moderate oligomerization. Strikingly, the Te@PDA‐Cu^II^ + NIR‐II group exhibited the most intense DLAT aggregation. Importantly, the enhanced DLAT oligomerization was significantly attenuated upon treatment with the copper‑specific chelator tetrathiomolybdate (TTM), directly establishing the Cu‑dependent nature of the observed proteotoxic stress (Figure 2F,G). Consistently, evaluation of upstream regulators FDX1 and LIAS revealed that both proteins were markedly depleted in Te@PDA‐Cu^II^ + NIR‐II‐treated cells (Figure 2H,I and Figure S11). Western blot analysis further corroborated these observations, showing distinct DLAT oligomer bands and coordinated downregulation of FDX1 and LIAS in Te@PDA‐Cu^II^ + NIR‐II treated group (Figure 2J). To confirm the predominant role of cuproptosis, MTT assays were performed in the presence of TTM. As shown in Figure S12, TTM treatment significantly reduced the cytotoxicity of Te@PDA‐Cu^II^ nanoreactors, with cell killing decreasing from 66.4% to 18.9%, confirming that the antitumor efficacy is primarily dependent on cuproptosis. Importantly, to demonstrate the broad generalizability and clinical relevance of the thermoelectric cascade‐enhanced cuproptosis, its induction by the Te@PDA‐Cu^II^ nanoreactors was further validated in the human breast cancer cell line MCF‐7. Consistent with the observations in 4T1 cells, confocal microscopy assays confirmed robust cuproptosis features in MCF‐7 cells, marked by significant DLAT aggregation and the down‐regulation of FDX1 and LIAS. Crucially, the introduction of TTM markedly reversed these cuproptosis‐associated protein alterations, thereby validating the highly versatile and broad applicability of our formulated nanoreactors in different breast cancer models. (Figure S13A–F)

Given that electron‐hole separation driven by the thermoelectric effect has been documented to promote radical generation, we also assessed the ROS‐producing capability of Te@PDA‐Cu^II^ under NIR‐II laser irradiation using 1,3‐diphenylisobenzofuran (DPBF) as a probe, where radical generation was evidenced by the time‐dependent decay of DPBF absorption (Figure S14). Then, the 2′,7′‐dichlorodihydrofluorescein diacetate (DCFH‐DA) was employed to evaluate intracellular ROS generation. As shown in Figure 2K, 4T1 cells treated with Te@PDA‐Cu^II^ under NIR‐II irradiation exhibited the strongest green fluorescence, indicating a marked ROS burst. Consistently, flow cytometric analysis quantitatively confirmed the significantly elevated ROS levels in this group (Figure 2L). As both intracellular ROS accumulation and cuproptosis can induce mitochondrial damage, we next used JC‐1 staining to assess mitochondrial membrane potential (MMP). Cells treated with Te@PDA‐Cu^II^ under NIR‐II laser irradiation exhibited dominant green fluorescence with minimal red signals, indicating severe MMP loss (Figure 2M and Figure S15). Collectively, these results demonstrate pronounced mitochondrial dysfunction induced by the thermoelectric effect. In addition, these combined effects also triggered cell apoptosis, as confirmed by Annexin V/PI flow cytometry. Cells in the Te@PDA‐Cu^II^ + NIR‐II group exhibited the highest proportion of early and late apoptotic populations compared with all control groups (Figure 2N,O).

Collectively, these results reveal that Te@PDA‐Cu^II^ acts as a sophisticated catalytic nanoreactor that exploits physical NIR‐II laser excitation to drive directional Cu^2+^ to Cu^+^ valence flip, thereby orchestrating a cascade‐enhanced cuproptosis effect within tumor cells.

### 2.3 Te@PDA‐Cu^II^ Thermoelectric Nanoreactors Induces Robust ICD Effect

**FIGURE 3:**
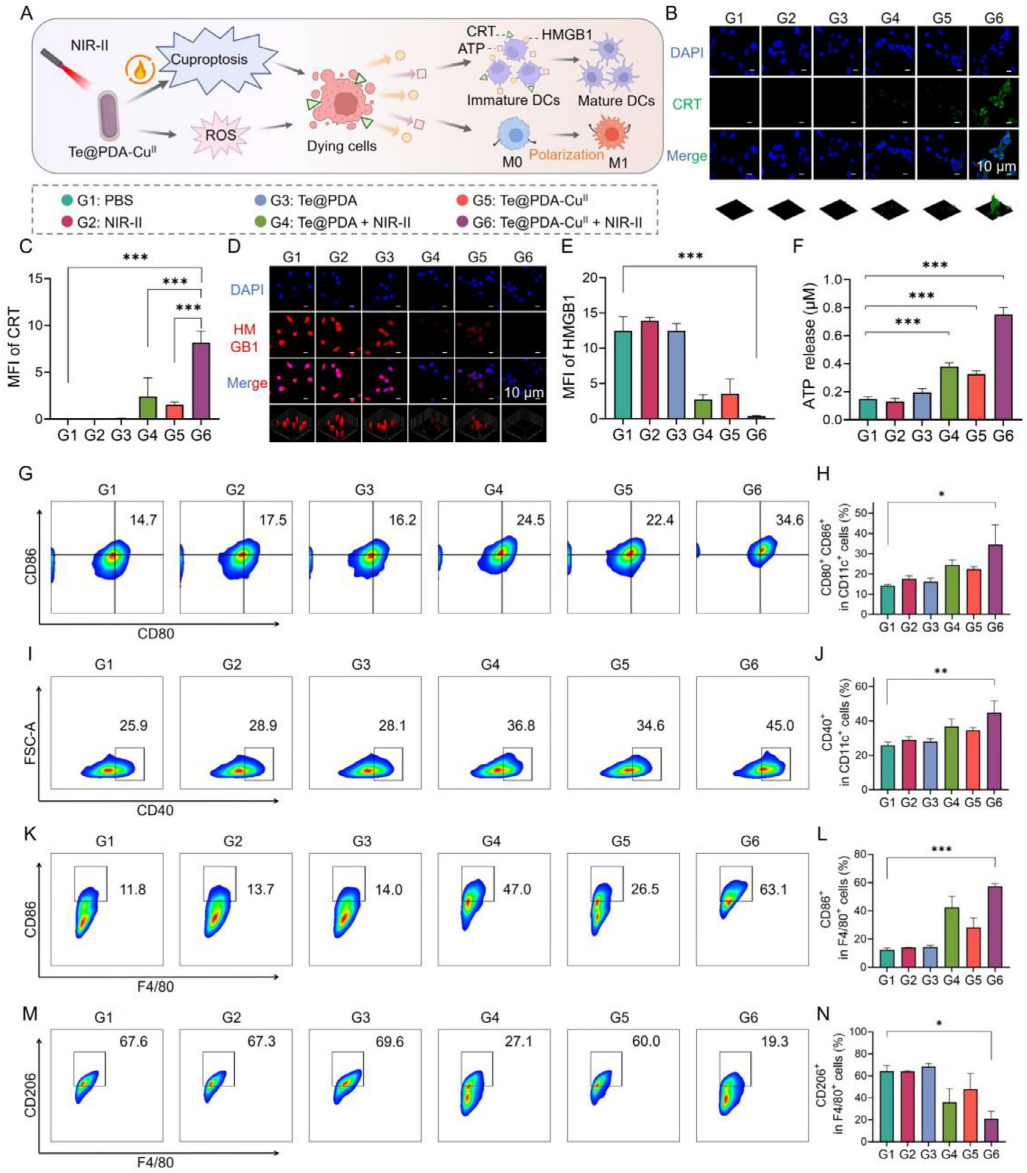
Te@PDA‐Cu^II^ thermoelectric nanoreactors induce robust ICD effect. (A) Schematic illustration of the ICD effect induced by Te@PDA‐Cu^II^ under NIR‐II laser irradiation. (B, C) Immunofluorescence images of CRT (B) and corresponding quantitative analysis of fluorescence intensity (C) in 4T1 cells following different treatments. (D, E) Immunofluorescence staining (D) and corresponding quantitative analysis (E) of HMGB1 in 4T1 cells after various treatments. (F) Detection of extracellular ATP secretion levels in 4T1 cells after different treatments. (G, H) Representative flow cytometric plots (G) and corresponding quantitative analysis (H) of DC maturation (CD80^+^CD86^+^) following co‐incubation with supernatants from 4T1 cells subjected to various treatments. (I, J) Representative flow cytometric plots of the co‐stimulatory molecule CD40 expressed on DCs (I) and corresponding quantitative analysis (J) after various treatments. (K, L) Representative flow cytometry plots (K) and corresponding quantitative analysis (L) of M1‐phenotype macrophages polarization (F4/80^+^CD86^+^) in BMDMs post various treatments. (M, N) Representative flow cytometry plots (M) and corresponding quantitative analysis (N) of M2‐phenotype macrophages polarization (F4/80^+^CD206^+^) in BMDMs post various treatments. Data are presented as mean ± SEM. The p‐values were determined using one‐way ANOVA followed by Tukey's post hoc test for multiple comparisons; ^*^ *p* < 0.05, ^**^ *p* < 0.01, ^***^ *p* < 0.001.

Following the confirmation of efficient cell killing effect via Te@PDA‐Cu^II^‐mediated thermoelectric cuproptosis, we further explored its capacity to trigger ICD, a critical step for activating systemic anti‐tumor immunity (Figure 3A). Immunofluorescence imaging was first employed to detect key hallmarks of ICD, including calreticulin (CRT) surface exposure and high mobility group box 1 (HMGB1) release. CRT exposure on the cell surface was negligible in the PBS, NIR‐II, or Te@PDA groups, while moderate CRT exposure was observed in the Te@PDA + NIR‐II (photothermal effect) and Te@PDA‐Cu^II^ (basal cuproptosis) groups. The Te@PDA‐Cu^II^ + NIR‐II group exhibited the most intense CRT fluorescence, indicating robust CRT exposure (Figure 3B,C). In parallel, the Te@PDA‐Cu^II^ + NIR‐II treatment induced the most substantial HMGB1 nuclear depletion (Figure 3D,E), further highlighting the potent immunogenicity elicited by enhanced cuproptosis. Meanwhile, the secretion of adenosine triphosphate (ATP), a vital DAMPs, was quantified using an ATP assay kit. As shown in Figure 3F, Te@PDA‐Cu^II^ + NIR‐II laser treatment could promote the most effective ATP release. Taken together, these results demonstrate that the NIR‐triggered thermoelectric catalytic cascade not only accelerates cuproptosis but also induces the release of DAMPs, thereby enabling a potent activation of the subsequent immune cascade. Moreover, MCF‐7 cells similarly exhibited a potent ICD effect upon nanoreactor treatment, characterized by enhanced CRT exposure, HMGB1 release, and extracellular ATP secretion (Figure S16A–E). These results definitively verify that our designed Te@PDA‐Cu^II^ nanoreactors possess extensive applicability across diverse tumor models.

Dendritic cells (DCs), acting as professional antigen‐presenting cells, recognize DAMPs and transition from an immature to a mature state, priming naïve T cells to initiate downstream antitumor immunity [21, 22]. To assess DC maturation, Te@PDA‐Cu^II^ + NIR‐II laser‐treated 4T1 cells were co‐cultured with mouse bone marrow‐derived dendritic cells (BMDCs). Flow cytometry revealed that the Te@PDA‑Cu^II^ + NIR‑II group induced the highest maturation rate (34.6%), significantly exceeding that of all other controls (Figure 3G,H). Concurrently, expression of CD40, a key co‐stimulatory molecule in antigen presentation, was markedly up‐regulated in the Te@PDA‑Cu^II^ + NIR‑II group (Figure 3I,J), pointing to enhanced potential for immune signal transduction. We then examined the phenotypic polarization of macrophages induced by Te@PDA‐Cu^II^ + NIR‐II irradiation using an indirect co‐culture system, wherein 4T1 cells were first treated with the nanoreactors under NIR‐II irradiation, followed by incubation of the harvested conditioned medium with mouse bone marrow‐derived macrophages (BMDMs). Flow cytometric analysis revealed that treatment with Te@PDA‐Cu^II^ combined with NIR‐II laser irradiation markedly promoted pro‐inflammatory M1‐type polarization, concurrently suppressing the M2‐type phenotype. Specifically, the proportion of the M1 marker CD86 surged from 12.4% in the PBS group to 57.5% in the Te@PDA‐Cu^II^ + NIR‐II group, while the M2 marker CD206 decreased sharply from 64.3% to 21.0% (Figure 3K–N). Together, these results indicate that under NIR‐II laser irradiation, Te@PDA‐Cu^II^ acts synergistically to both amplify cuproptosis and induce a potent ICD effect, promoting DC maturation and pro‐inflammatory macrophage polarization.

### 2.4 Te@PDA‐Cu^II^ Thermoelectric Nanoreactors Inhibit Breast Cancer Growth and Activate Anti‐tumor Immunity

**FIGURE 4:**
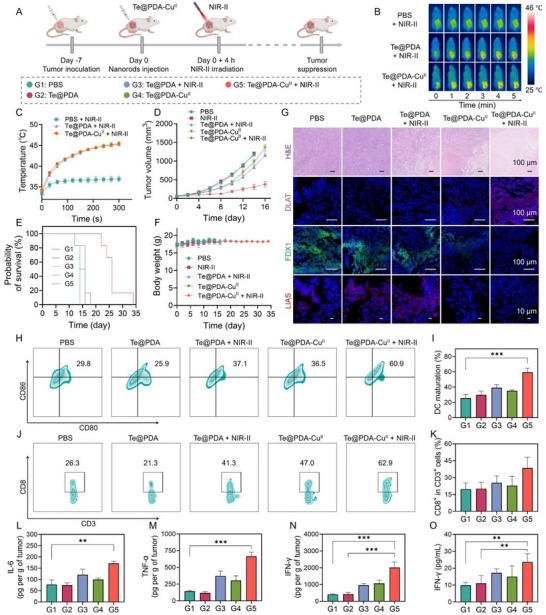
Te@PDA‐Cu^II^ thermoelectric nanoreactors inhibit breast cancer growth and activate anti‐tumor immunity. (A) Schematic illustration of the therapeutic regimen for 4T1 subcutaneous tumor models. (B, C) IR thermal images of tumor‐bearing mice exposed to the NIR‐II laser irradiation for different durations (B) and the corresponding heating curves (C). (D) Tumor growth curves of 4T1 tumor‐bearing mice receiving different treatments. (E) Survival curves of mice in the various treatment groups. (F) Body weight changes of mice during the treatment period. (G) Representative H&E staining images of tumor tissues and immunofluorescence staining of DLAT, FDX1, and LIAS in the various treatment groups. (H, I) Flow cytometric plots (H) and corresponding quantitative analysis (I) of matured DCs within the tumor post various treatments. (J, K) Flow cytometric plots (J) and corresponding quantitative analysis (K) of tumor‐infiltrating CD8^+^ T cells within the tumor post various treatments. (L–N) Intratumoral levels of IL‐6 (L), TNF‐α (M), and IFN‐γ (N) measured by ELISA. (O) Serum levels of IFN‐γ were detected after different treatments. Data are presented as mean ± SEM. The p‐values were determined using one‐way ANOVA followed by Tukey's post hoc test for multiple comparisons; ^*^ *p* < 0.05, ^**^ *p* < 0.01, ^***^ *p* < 0.001.

Before assessing the therapeutic efficacy in vivo, the hemocompatibility of Te@PDA‑Cu^II^ was first examined through an ex vivo hemolysis assay. Co‑incubation with fresh whole blood at concentrations up to 200 µg/mL yielded a hemolysis rate below 5%, demonstrating its favorable biosafety for further in vivo use (Figure S17). Then, we carefully evaluated the in vivo anticancer efficacy of the Te@PDA‑Cu^II^ thermoelectric nanoreactors using a subcutaneous 4T1 murine tumor model (Figure 4A). We first assessed the photothermal performance of intratumorally injected Te@PDA‑Cu^II^ under NIR‑II laser irradiation via infrared thermal imaging. As shown in Figure 4B,C, while the PBS group showed only a minor temperature increase (ΔT ≈ 3.4°C), both the Te@PDA and Te@PDA‑Cu^II^ groups exhibited pronounced local hyperthermia (ΔT > 11.1 °C). Such efficient photothermal conversion establishes the necessary temperature gradient to drive thermoelectric catalysis, providing the physical basis for the subsequent cascade‑catalytic processes. To evaluate the antitumor efficacy of the Te@PDA‑Cu^II^ thermoelectric nanoreactors, subcutaneous 4T1 tumor‑bearing mice were randomly divided into five groups: PBS, NIR‑II, Te@PDA + NIR‑II, Te@PDA‑Cu^II^, and Te@PDA‑Cu^II ^+ NIR‑II. On day 0, the respective agents were administered via intratumoral injection, after which the designated groups received NIR‑II laser irradiation. As shown in Figure 4D,E, 4T1 tumors in the PBS and NIR‑II groups displayed a rapid growth trend. While the Te@PDA + NIR‐II (photothermal only) and Te@PDA‐Cu^II^ (cuproptosis) groups exhibited moderate growth inhibition. In striking contrast, the Te@PDA‐Cu^II^ + NIR‐II group achieved the most potent tumor suppression and significantly prolonged overall survival. Notably, the body weights of all mice remained stable throughout the experiment (Figure 4F). H&E staining of tumor sections further confirmed that the Te@PDA‐Cu^II^ + NIR‐II treatment induced the most substantial necrosis and structural disruption in tumor tissues (Figure 4G). Meanwhile, through immunofluorescence analysis, we observed that the Te@PDA‐Cu^II^ + NIR‐II group exhibited the most effective upregulation of DLAT, along with a concurrent downregulation of FDX1 and LIAS, indicating a robust induction of copper‐dependent cell death (Figure 4G and Figure S18).

Subsequently, to investigate the underlying immunological mechanisms of the Te@PDA‐Cu^II^ thermoelectric effect on tumor growth inhibition, we first evaluated its capacity to induce ICD. Immunofluorescence staining of tumor tissues revealed that the Te@PDA‐Cu^II^ + NIR‐II laser irradiation treatment elicited the most potent ICD, as evidenced by intense surface exposure of CRT and significant release of HMGB1 from the nucleus (Figure S19). On day 5 post‐treatment, tumors were harvested, and single‐cell suspensions were prepared for staining and flow cytometric analysis. The results showed that Te@PDA‐Cu^II^ + NIR‐II irradiation treatment promoted the highest level of DC maturation, with the percentage of CD80^+^CD86^+^ cells increasing from 25.6% (PBS) to 59.5% (Figure 4H,I). Concurrently, flow cytometric analysis also revealed a significant repolarization of tumor‐associated macrophages (TAMs) toward the pro‐inflammatory M1 phenotype, with the proportion of F4/80^+^CD80^+^ cells rising from 39.0% to 75.0% (Figure S20). Importantly, the proportion of tumor‐infiltrating cytotoxic T cells (CD3^+^CD8^+^) rose sharply from 20.2% (PBS) to 40.7% in the Te@PDA‐Cu^II^ + NIR‐II irradiation treatment group (Figure 4J,K). Such enhanced recruitment of effector T cells was further supported by elevated levels of Granzyme B (from 24.2% to 56.6%) and IFN‐γ (from 26.4% to 60.0%) on CD8^+^ T cells (Figure S21). The expression levels of antitumor‑associated cytokines were further quantified in both tumor tissues and serum by Enzyme‐Linked Immunosorbent Assay (ELISA). Notably, compared to all other groups, the Te@PDA‑Cu^II^ + NIR‑II irradiation group exhibited significantly elevated intratumoral secretion of TNF‑α, IFN‑γ, and IL‑6, along with increased serum levels of IFN‑γ (Figure 4L–O).

Overall, the Te@PDA‑Cu^II^‐mediated thermoelectric effect treatment effectively suppressed tumor growth by inducing potent ICD, promoting DC maturation and cytotoxic T‑cell infiltration, and enhancing the secretion of key antitumor cytokines, thereby orchestrating a robust antitumor immune response.

### 2.5 Te@PDA‐Cu^II^ Thermoelectric Nanoreactors Effectively Suppress the Growth and Metastasis of Orthotopic Breast Cancer

**FIGURE 5:**
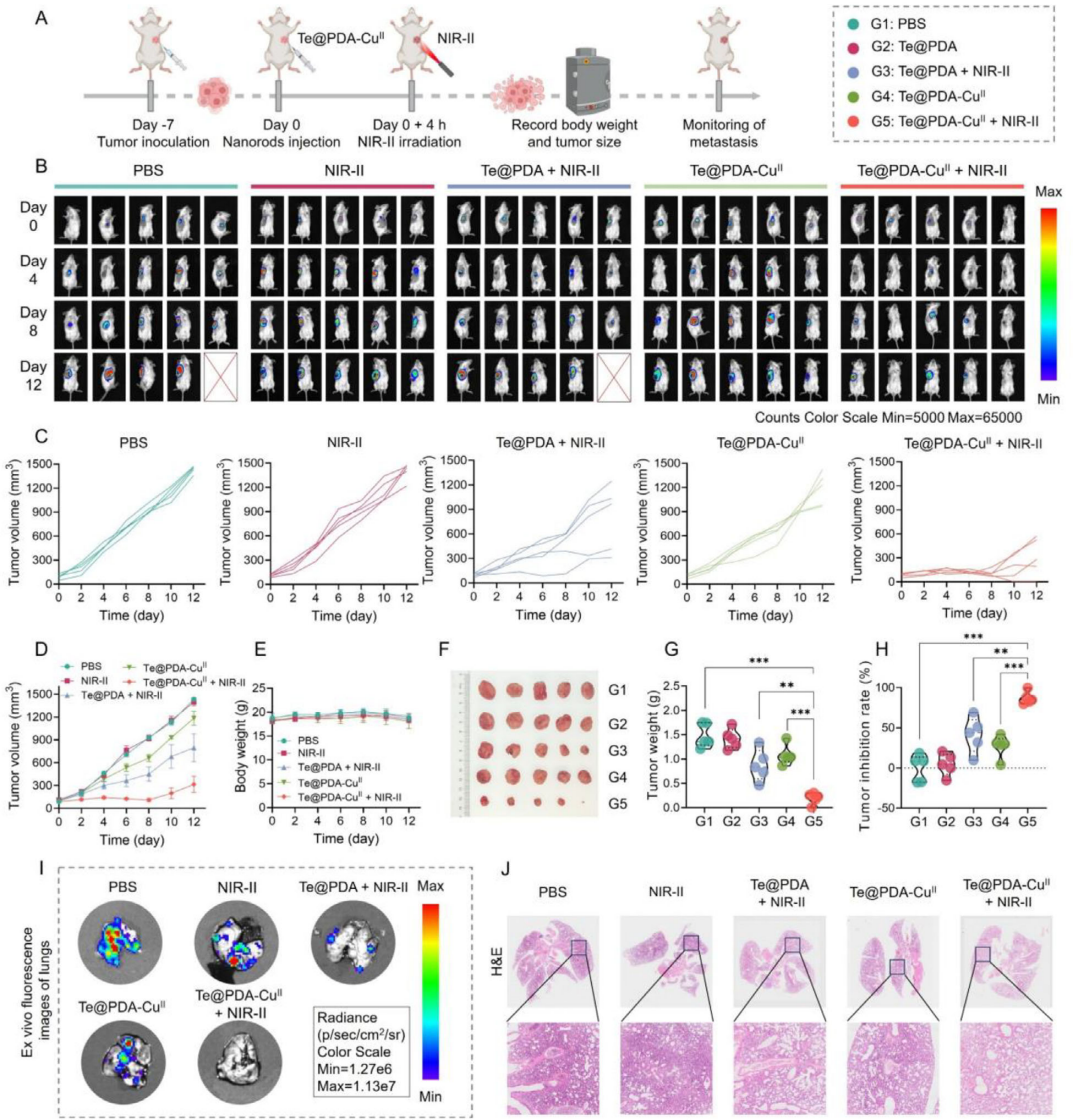
Te@PDA‐Cu^II^ thermoelectric nanoreactors effectively suppress the growth and metastasis of orthotopic breast cancer. (A) Schematic illustration of the therapeutic regimen for orthotopic 4T1‐Luci tumor growth and lung metastasis. (B) Longitudinal bioluminescence imaging of 4T1‐Luci tumor in different treatment groups using an in vivo imaging system. (C, D) Individual (C) and average (D) tumor growth curves of 4T1‐Luci tumor‐bearing mice receiving different treatments. (E) Body weight changes of mice were monitored during the treatment period. (F) Photographs of representative excised tumors at the endpoint. (G, H) Average tumor weights (G) and tumor inhibition rates (H) were calculated at the end of the treatment. (I) Ex vivo bioluminescence imaging of lungs harvested at the treatment endpoint to evaluate metastasis. (J) Representative H&E‐stained histological sections of lung tissues. Data are presented as mean ± SEM. The p‐values were determined using one‐way ANOVA followed by Tukey's post hoc test for multiple comparisons; ^*^ *p* < 0.05, ^**^ *p* < 0.01, ^***^ *p* < 0.001.

Compared to conventional subcutaneous xenografts, orthotopic breast cancer models more faithfully recapitulate the hostile tumor microenvironment and display highly aggressive and metastatic behavior [23, 24]. Given the potent antitumor efficacy and systemic immune activation elicited by Te@PDA‑Cu^II^ under NIR‐II laser irradiation, we established a luciferase‑labeled orthotopic 4T1 (4T1‐Luci) tumor model to evaluate its ability to suppress primary tumor growth and prevent pulmonary metastasis (Figure 5A). As depicted in the bioluminescence images and tumor growth curves (Figure 5B–D), tumors in the PBS and NIR‑II laser irradiation groups displayed uncontrolled proliferation throughout the 12‑day observation period. While the Te@PDA + NIR‑II (photothermal therapy) and Te@PDA‑Cu^II^ (cuproptosis) groups showed moderate growth inhibition. In striking contrast, the Te@PDA‑Cu^II^ + NIR‑II group achieved the most potent suppression of orthotopic 4T1 tumor growth. Importantly, body weight remained stable in all groups, indicating no appreciable systemic toxicity following treatment (Figure 5E). Following the treatment, orthotopic tumors were excised, photographed, and weighed. Representative images visually confirmed the smallest tumor size in the Te@PDA‑Cu^II^ + NIR‑II group (Figure 5F). Consistent with this observation, the average tumor weight in this group was significantly lower than that in all other groups (Figure 5G). Based on tumor weight, the Te@PDA‑Cu^II^ + NIR‑II group achieved a tumor inhibition rate of 84.8%, which was substantially higher than those in the monotherapy groups (Te@PDA + NIR‑II: 51.2%; Te@PDA‑Cu^II^: 28.8%) (Figure 5H).

Since the lung is the predominant site of distant metastasis in breast cancer, we then evaluated pulmonary metastasis. After luciferin injection and lung dissection, bioluminescence imaging showed obvious fluorescence signals in the PBS and NIR‑II groups, reflecting a high level of metastasis. In contrast, only negligible bioluminescence was detected in the Te@PDA‑Cu^II^ + NIR‑II group (Figure 5I). Histological analysis with H&E staining further confirmed these observations at the microscopic level, in which no metastatic nodules were observed in the Te@PDA‑Cu^II^ + NIR‑II group (Figure 5J). Collectively, these results demonstrate that the Te@PDA‑Cu^II^‑enabled thermoelectric enhancement of cuproptosis not only effectively suppresses the growth of orthotopic 4T1 tumors but also potently inhibits pulmonary metastasis.

### 2.6 Te@PDA‐Cu^II^ Thermoelectric Nanoreactors Synergize With ICB to Elicit Abscopal Effects in Bilateral Tumor Models

**FIGURE 6:**
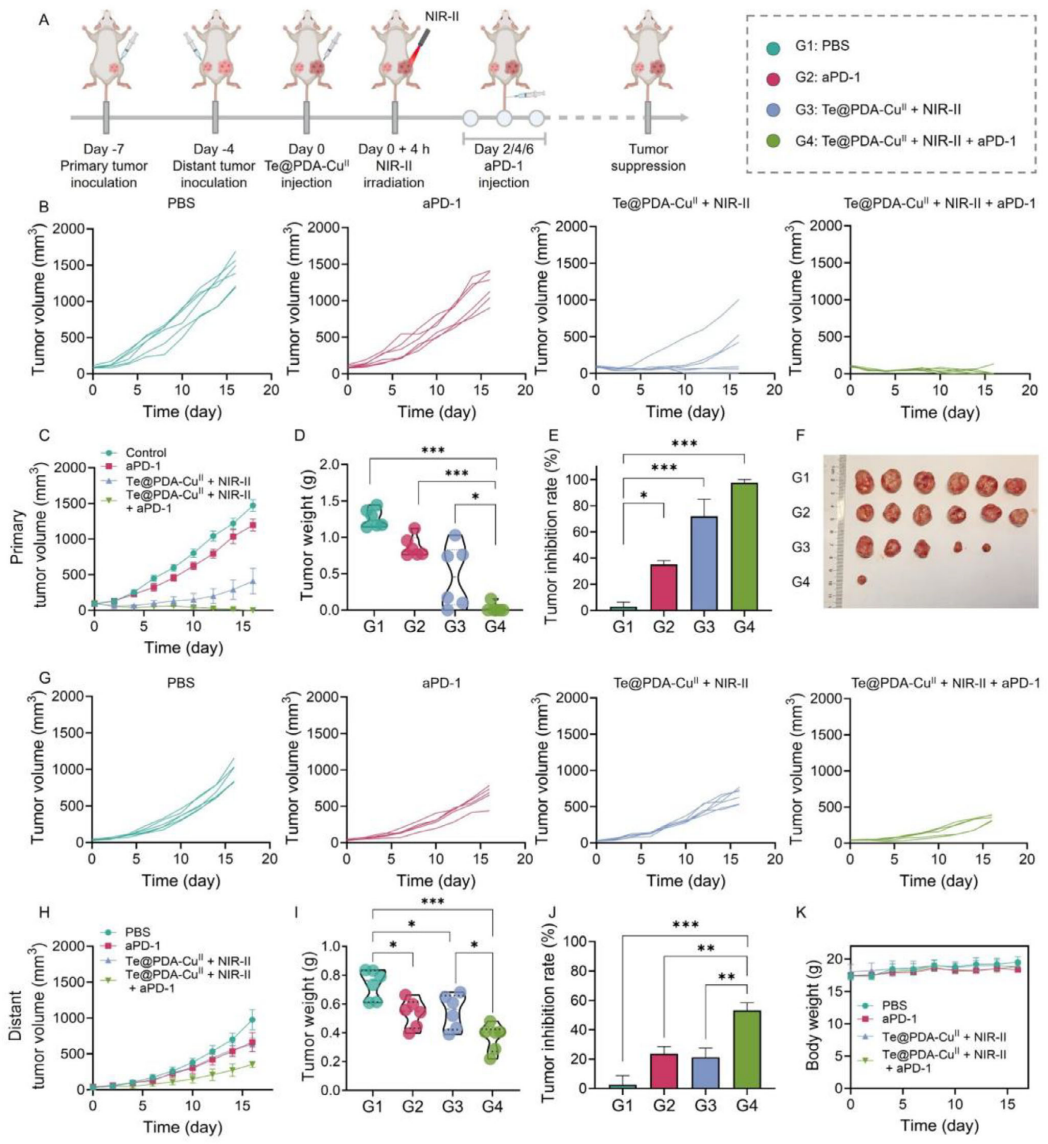
Te@PDA‐Cu^II^ thermoelectric nanoreactors synergize with ICB to elicit abscopal effects in bilateral tumor models. (A) Schematic diagram of bilateral 4T1 tumor model establishment and combined aPD‐1 therapy protocol. (B, C) Individual (B) and average (C) tumor growth curves of the primary tumors (treated side) after different treatments. (D, E) Average tumor weights (D) and tumor inhibition rates (E) of the primary tumors at the endpoint in different treatment groups. (F) Representative photographs of primary tumors after different treatments. (G, H) Individual (G) and average (H) tumor growth curves of the distant tumors (untreated side) after different treatments. (I, J) Average tumor weights (I) and tumor inhibition rates (J) of the distant tumors at the endpoint in different treatment groups. (K) Body weight changes of mice after various treatments. Data are presented as mean ± SEM. The p‐values were determined using one‐way ANOVA followed by Tukey's post hoc test for multiple comparisons; ^*^ *p* < 0.05, ^**^ *p* < 0.01, ^***^ *p* < 0.001.

Motivated by the excellent the pronounced tumor suppression and immune activation observed in our preliminary studies, we further established a bilateral 4T1 tumor model to investigate whether the combination of Te@PDA‑Cu^II^ thermoelectric nanoreactors with immune checkpoint blockade (ICB) could elicit abscopal effects (Figure 6A). In the established bilateral tumor model, primary tumors receiving the combination of Te@PDA‑Cu^II^ + NIR‑II irradiation and aPD‑1 antibody exhibited the most effective tumor growth suppression (Figure 6B,C), the lowest final tumor weight, and the highest tumor inhibition rate (∼97.6%) (Figure 6D–F). Notably, the untreated distal tumors in the same mice also showed significant growth retardation (Figure 6G,H), reduced weight, and a marked inhibition rate (∼53.3%) (Figure 6I,J), indicating a potent abscopal effect. Throughout the treatment, all groups maintained stable body weight, suggesting no significant systemic toxicity (Figure 6K). Therefore, the combination of Te@PDA‑Cu^II^ + NIR‑II irradiation and aPD‑1 antibody not only potently inhibits primary tumor growth but also elicits a robust abscopal effect, significantly suppressing distal tumors without inducing systemic toxicity in a bilateral 4T1 model.

### 2.7 Te@PDA‐Cu^II^ Thermoelectric Nanoreactors Synergize With ICB to Establish Long‐Term Anti‐Tumor Immune Memory

**FIGURE 7:**
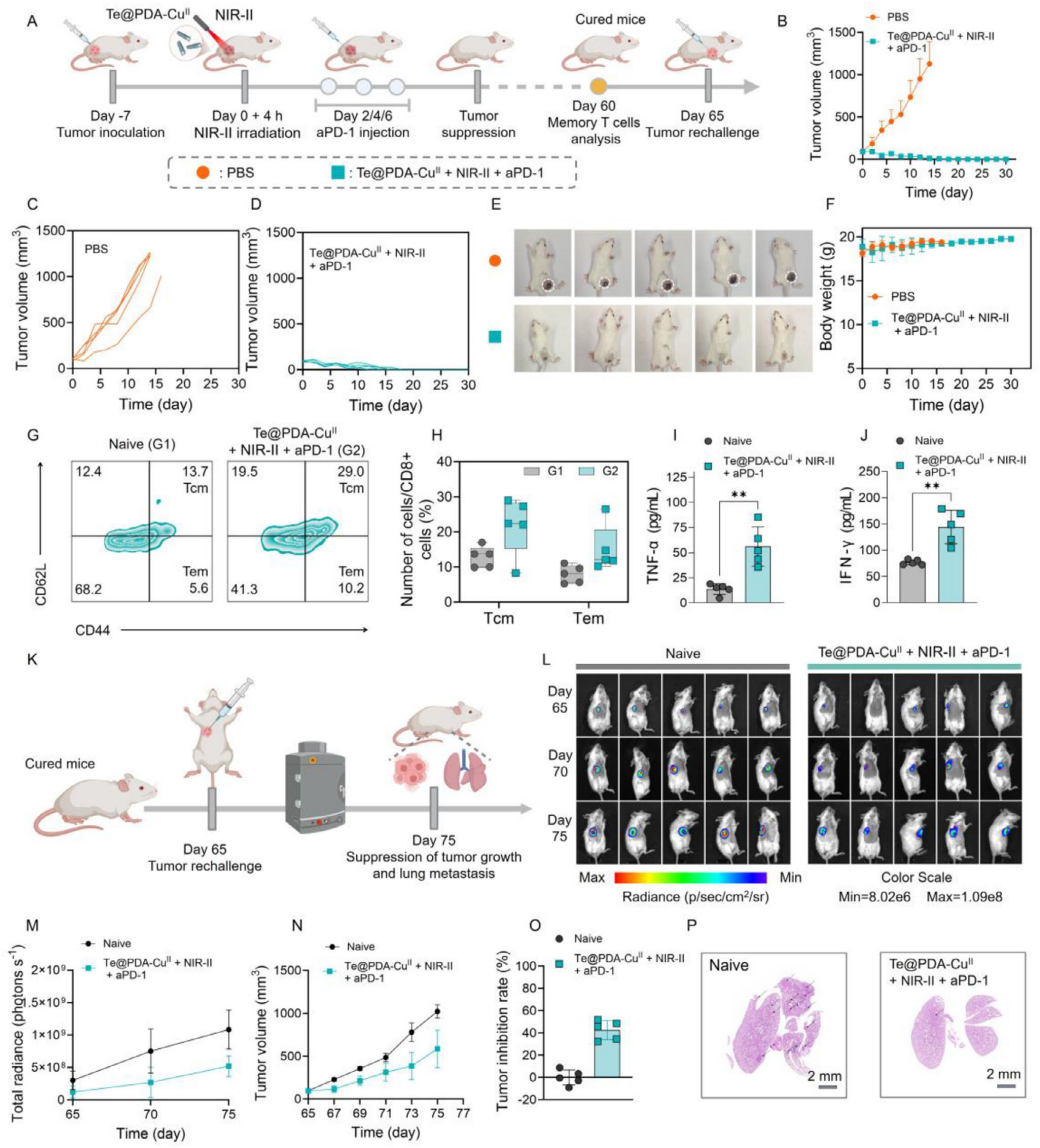
Te@PDA‐Cu^II^ thermoelectric nanoreactors synergize with ICB to establish long‐term anti‐tumor immune memory. (A) Schematic illustration of the experimental regimen for evaluating the long‐term immune memory on a 4T1 tumor model. (B–D) Average (B) and individual (C, D) tumor growth curves of subcutaneous 4T1 tumors in mice after various treatments. (E) Representative photographs of tumor‐bearing mice after various treatments. (F) Body weight changes of mice were monitored during the treatment period. (G, H) Flow cytometric plots (G) and corresponding quantitative analysis (H) of central memory T cells (Tcm, CD8^+^CD44^+^CD62L^+^) and effector memory T cells (Tem, CD8^+^CD44^+^CD62L^−^) in the peripheral blood of cured mice compared to naive controls. (I, J) Serum levels of TNF‐α (I) and IFN‐γ (J) in cured mice compared to naive controls. (K) Schematic illustration of the orthotopic 4T1‐Luci tumor re‐challenge model and lung metastasis inhibition. (L, M) In vivo bioluminescence imaging (L) and quantification (M) of orthotopic 4T1‐Luci tumor growth in naive and cured mice following re‐challenge. (N) Tumor growth curves of naive (control) and cured mice following re‐challenge. (O) Tumor inhibition rates in the re‐challenge experiment. (P) Representative H&E staining of lung tissues evaluating metastatic nodules. Data are presented as mean ± SEM. The p‐values were determined using one‐way ANOVA followed by Tukey's post hoc test for multiple comparisons; ^*^ *p* < 0.05, ^**^ *p* < 0.01, ^***^ *p* < 0.001.

To further assess whether the combination of Te@PDA‐Cu^II^ + NIR‐II and aPD‐1 antibody elicits long‐term immunological memory, we established a unilateral 4T1 subcutaneous tumor model (Figure 7A). The results showed that the combination treatment achieved complete tumor eradication in the 4T1 model, while no significant changes in body weight were observed across groups (Figure 7B–F and Figure S22), indicating good biosafety. At day 60 post‐treatment, peripheral blood was collected from cured mice for immune memory analysis. Flow cytometry revealed that the proportion of central memory T cells (Tcm, CD8^+^CD44^+^CD62L^+^) increased from 12.8% in naïve mice to 21.8% in the Te@PDA‐Cu^II^ + NIR‐II + aPD‐1 group, while effector memory T cells (Tem, CD8^+^CD44^+^CD62L^−^) rose from 7.9% to 15.1% (Figure 7G,H), suggests the establishment of long‐term anti‐tumor immune memory capacity. Serum ELISA further showed significantly elevated levels of the pro‐inflammatory cytokines TNF‐α and IFN‐γ in the combination group compared to negative controls (Figure 7I,J).

On day 65, both naive mice and cured mice from the combination group were orthotopically re‐challenged with 4T1‐Luci tumors in the mammary fat pad (Figure 7K). In vivo imaging showed markedly weaker tumor bioluminescence in cured mice relative to naive controls (Figure 7L,M). Correspondingly, tumor growth curves demonstrated rapid progression in control mice, whereas cured mice exhibited potent tumor suppression with an inhibition rate of ∼42.6% (Figure 7N,O and Figure S23). H&E staining revealed abundant metastatic nodules in the lungs of control mice, whereas the cured group showed ignored detectable metastases (Figure 7P and Figure S24).

Collectively, these results demonstrate that the NIR laser‑triggered thermoelectric cascade catalysis mediated by Te@PDA‑Cu^II^, in synergy with aPD‑1 blockade, not only achieves localized tumor eradication but also establishes systemic anti‑tumor immune memory to effectively suppress local tumor recurrence and inhibit distant pulmonary metastasis.

Finally, to evaluate the comprehensive long‐term biocompatibility of Te@PDA‐Cu^II^, we performed hematological and serum biochemical analyses on days 1 and 10 post‐administration. The results revealed that hepatic function markers (e.g., AST) and renal function indicators (e.g., Cr) in the treated mice remained stable and comparable to those of the control group. Furthermore, hematological assessments indicated no significant abnormalities in blood cell parameters relative to the control (Figure S25). Collectively, these results confirm the excellent in vivo biocompatibility of Te@PDA‐Cu^II^ at the tested dosage.

## 3 Discussion

Cuproptosis has recently been recognized as a copper‐dependent form of regulated cell death with unique metabolic vulnerability. However, its translational potential has been largely constrained by the lack of precise control over intracellular Cu^+^ availability [3]. In most reported strategies, exogenous copper salts or ionophores are employed, which inevitably suffer from poor tumor specificity, systemic toxicity, and inefficient Cu^2+^‐to‐Cu^+^ conversion within the tumor microenvironment [25]. In this work, we address this long‐standing bottleneck by introducing a NIR‐II laser‐activated thermoelectric nanoreactor capable of supplying electrons in situ to drive directional copper valence conversion. By integrating Te nanorods with a conductive PDA interlayer, Te@PDA‐Cu^II^ effectively translates externally applied physical energy into localized biochemical redox regulation. Upon NIR‐II laser irradiation, the thermoelectric response of Te generates a sustained electron flux that is efficiently extracted and relayed by PDA to reduce chelated Cu^2+^ into bioactive Cu^+^, thereby directly overcoming the valence limitation that has impeded the practical exploitation of cuproptosis.

Mechanistically, the thermoelectrically amplified Cu^+^ pool initiates a cascade of copper‐dependent proteotoxic stress, characterized by DLAT oligomerization, destabilization of Fe‐S cluster proteins, and collapse of mitochondrial metabolism. Notably, this mode of cell death extends beyond metabolic catastrophe and evolves into a highly immunogenic process. The concurrent ROS burst, mitochondrial dysfunction, and extensive proteome stress collectively promote robust immunogenic cell death, as evidenced by CRT exposure, HMGB1 release, and ATP secretion. These danger signals orchestrate the maturation of dendritic cells and reshape innate immune polarization by favoring pro‐inflammatory M1 macrophages over immunosuppressive M2 phenotypes. Mechanistically, the M1 polarization of TAMs is an indirect effect mediated by DAMPs (e.g., HMGB1 and ATP) released from cuproptotic tumor cells, rather than a direct effect of the nanoreactors on these immune cells. It is plausible that these DAMPs engage canonical pattern recognition receptors (such as TLR4) on macrophages, potentially triggering downstream pro‐inflammatory signaling cascades. Importantly, this immune activation is not merely a secondary consequence of tumor ablation but is intrinsically coupled to the thermoelectric‐driven cuproptosis cascade, establishing a direct link between physical energy conversion, metal redox chemistry, and adaptive immune priming.

In vivo, such NIR‐II laser‐initiated thermoelectric‐immunological cascade translates into robust therapeutic outcomes in various breast cancer models. Te@PDA‐Cu^II^ under NIR‐II laser irradiation achieves efficient ablation of primary tumors, suppresses pulmonary metastasis in orthotopic settings, and, when combined with aPD‐1 blockade, elicits pronounced abscopal effects in bilateral tumor models. More importantly, the combination therapy establishes durable immune memory, conferring long‐term protection against tumor rechallenge and metastatic relapse. These results highlight the advantages of our Te@PDA‐Cu^II^ thermoelectric nanoreactors: thermoelectric nanomaterials can function as programmable bioelectronic interfaces that transduce non‐invasive NIR‐II light energy into precise intracellular redox signals, thereby coupling metal‐dependent cell death with adaptive immunity. Beyond cuproptosis, this physical‐energy‐driven strategy may be extended to regulate other redox‐sensitive pathways and synergize with immunotherapy, radiotherapy, or metabolic interventions that are otherwise limited by insufficient intratumoral control.

However, the current study still has several limitations. First, the in vivo antitumor efficacy of Te@PDA‐Cu^II^ was evaluated only via intratumoral injection in a subcutaneous breast cancer model, which does not reflect clinically relevant systemic administration. Whether this nanoreactor can reach tumors through the bloodstream remains unknown. Second, the subcutaneous tumor model does not capture the complexity of the tumor microenvironment or metastatic disease. Moreover, although the study confirmed the activation of antitumor immunity, the changes in immunosuppressive cells, including regulatory T cells (Tregs) and myeloid‐derived suppressor cells (MDSCs), were not analyzed. Consequently, the mechanism of immune activation and tumor microenvironment remodeling remains incomplete. Third, toxicity data from intratumoral injection do not predict the safety profile following systemic delivery.

To address these limitations, future studies should evaluate the systemic delivery of Te@PDA‐Cu^II^ via intravenous injection, including biodistribution, tumor accumulation, and therapeutic efficacy. Surface modifications (e.g., PEGylation or tumor‐targeting ligands) may be explored to enhance circulation time and tumor specificity. Additionally, comprehensive immunoprofiling of immunosuppressive populations should be incorporated to fully understand the impact on the tumor immune microenvironment. Finally, comprehensive toxicological assessments following systemic administration are warranted to support translational development.

## 4 Conclusions

In summary, our study demonstrates a novel breast cancer treatment strategy centered on a NIR‐II laser‐initiated thermoelectric‐immunological cascade. Under NIR‐II irradiation, Te NRs generate a thermoelectric electron flow that is directionally relayed via the PDA interlayer to reduce Cu^2+^ to Cu^+^, igniting potent cuproptosis and effectively suppresses tumor proliferation. Moreover, the Te@PDA‐Cu^II^ thermoelectric nanoreactors further trigger robust ICD, releasing DAMPs that promote dendritic‐cell maturation and cytotoxic T‐cell infiltration. In bilateral and rechallenge models, coupling this cascade with aPD‐1 checkpoint blockade not only inhibits primary and distal tumor growth but also establishes long‐term anti‐tumor immune memory, effectively preventing recurrence and pulmonary metastasis. Together, our study highlights a physical‐energy‑driven, cuproptosis‑mediated, and immunologically amplified strategy for synergistic and systemic breast cancer therapy.

## 5 Experimental Section

### 5.1 Materials

Sodium tellurite (Na_2_TeO_3_), ascorbic acid, polyvinylpyrrolidone (PVP), and cupric chloride dihydrate were purchased from Sinopharm Chemical Reagent Co., Ltd., China. Dopamine was purchased from J&K Scientific Co., Ltd., China. 3‐(4,5‐Dimethylthiazol‐2‐yl)‐2,5‐ diphenyltetrazolium bromide (MTT) was obtained from Macklin Co., Ltd., China. Annexin V‐FITC apoptosis detection kit was sourced from YiFeiXue Biotechnology Co., Ltd., China. Mitochondrial membrane potential assay kit with JC‐1 and ATP assay kit were purchased from Beyotime Co., Ltd., China. Antibodies for flow cytometry including anti‐CD11C‐FITC (clone N418, catalog: 117 305), anti‐CD40‐PE‐Cy7 (clone 3/23, catalog: 124 621), anti‐CD80‐APC (clone 16‐10A1, catalog: 104 713), anti‐CD86‐PE (clone GL‐1, catalog: 105 007), anti‐CD206‐APC (clone C068C2, catalog: 141 708), anti‐CD3‐APC‐Cy7 (clone 17A2, catalog: 100 222), anti‐CD8‐PerCP (clone 53–6.7, catalog: 100 731), anti‐CD45‐BV650 (clone 30‐F11, catalog: 103 151), anti‐IFN‐γ‐PE‐Cy7 (clone W18272D, catalog: 163 507), anti‐CD3‐FITC (clone 17A2, catalog: 100 203), anti‐CD44‐APC (clone IM7, catalog: 103 011), anti‐ CD45‐BV605 (clone S18009F, catalog: 157 217), and anti‐CD62L‐PE‐Cy7 (clone  MEL‐14, catalog: 104 417), were obtained from Biolegend. All ELISA kits were purchased from Thermo Fisher Scientific. Antibodies for immunofluorescence staining, including DLAT polyclonal antibody (Cat No. 13 426‐1‐AP), FDX1 polyclonal antibody (Cat No. 12 592‐1‐AP), LIAS polyclonal antibody (Cat No. 11 577‐1‐AP), HMGB1 polyclonal antibody (Cat No. 10 829‐1‐AP), and calreticulin polyclonal antibody (Cat No. 10 292‐1‐AP), were purchased from Proteintech.

### 5.2 Synthesis of Te@PDA‐Cu^II^ Thermoelectric Nanoreactors

Tellurium nanorods (Te NRs) were synthesized via a modified hydrothermal reduction method according to previous literature. Briefly, 20 mg of sodium metatellurite (Na_2_TeO_3_) and 1 g of ascorbic acid were dissolved in 40 mL of an aqueous PVP solution (20 mg mL^−1^) under magnetic stirring until a homogeneous mixture was obtained. The resulting solution was transferred into a sealed Teflon‐lined stainless‐steel autoclave and maintained at 90°C for 20 h. After the reaction was complete and the system had cooled to room temperature, the initial Te NRs product was collected by centrifugation (10000 rpm, 5 min), followed by repeated washing with ethanol and deionized water.

To coat the Te NRs with a polydopamine layer, the as‐prepared Te NRs (1 mg mL^−1^) were dispersed in an aqueous dopamine solution (1 mg mL^−1^) under continuous stirring. The pH of the mixture was adjusted to 8.5 using Tris‐HCl buffer (10 mM), and the reaction was allowed to proceed for 2 h at room temperature. The polydopamine‐coated Te NRs (denoted as Te@PDA) were then isolated by centrifugation at 10000 rpm for 5 min and washed with deionized water.

For the chelation of copper ions, a predetermined amount of copper chloride (10 mM) was added dropwise to an aqueous dispersion of Te@PDA (1 mg mL^−1^) under vigorous stirring. The mixture was stirred magnetically for 90 min at room temperature to ensure sufficient coordination. The final product, designated as Te@PDA‐Cu^II^, was harvested by centrifugation at 10000 rpm for 5 min, washed thoroughly with deionized water, and stored at 4°C for further use.

### 5.3 Characterization

The microstructure and elemental distribution of the Te@PDA‐Cu^II^ nanorods were characterized using transmission electron microscopy (TEM, Talos 200X, Sigma) equipped with energy‐dispersive X‐ray spectroscopy (EDS). Variations in the zeta potential of different intermediate products were measured using a Malvern Zetasizer Pro. The elemental composition and chemical states of Te@PDA‐Cu^II^ were analyzed by X‐ray photoelectron spectroscopy (XPS, Thermo Scientific K‐Alpha). The crystal structure of the samples was examined using an X‐ray diffractometer (XRD, SmartLab SE). Surface functional groups were identified via Fourier transform infrared spectroscopy (FTIR, INVENIO S), and the absorbance of the samples was recorded using a UV–vis‐NIR spectrophotometer (Genesys 50). To evaluate photothermal conversion performance, real‐time temperature changes were monitored with an infrared thermal imaging camera. The photoresponse current (i‐t), photothermoelectric potential (V‐t), and electrochemical impedance spectroscopy (EIS) were measured using an electrochemical workstation.

### 5.4 Photothermal Performance of Te@PDA‐Cu^II^ Under NIR‐II Laser Irradiation

The photothermal performance of Te@PDA‐Cu^II^ was evaluated by irradiating aqueous dispersions with a 1064 nm NIR‐II laser and monitoring the temperature in real‐time using an infrared thermal imaging camera. First, to assess concentration‐dependent heating, samples at concentrations of 0, 25, 50, 100, and 200 µg mL^−1^ were exposed to a fixed laser power density of 0.6 W cm^−2^ for 10 min. Moreover, photothermal stability was tested over three consecutive on/off cycles, where each cycle comprised 5 min of irradiation (0.6 W cm^−2^) and 10 min of cooling.

### 5.5 Thermoelectric Properties of Te@PDA‐Cu^II^ Thermoelectric Nanoreactors

The electrochemical performance of Te@PDA‐Cu^II^ was assessed using a standard three‐electrode system on a CHI 760E electrochemical workstation (Shanghai Chenhua). First, a uniform layer of Te@PDA‐Cu^II^ was deposited onto a fluorine‐doped tin oxide (FTO) glass substrate via electrodeposition to serve as the working electrode. The electrochemical cell was then assembled by immersing the working electrode, along with a graphite counter electrode and a saturated calomel reference electrode, into a 0.1 M Na_2_SO_4_ electrolyte solution. Under irradiation with a 1064 nm NIR‐II laser, real‑time changes in photoresponse current and photoresponse potential were monitored to verify the thermoelectric behavior. Electrochemical impedance spectroscopy was further conducted to investigate interfacial charge‑transfer kinetics.

### 5.6 Detection of Cu^+^ Generation From Te@PDA‐Cu^II^ Under NIR‐II Laser Irradiation

To quantify the change in Cu^+^/Cu^2+^ ratio induced by thermoelectric conversion, XPS was performed on Te@PDA‑Cu^II^ before and after NIR‑II laser irradiation. Briefly, Te@PDA‑Cu^II^ were irradiated with a 1064 nm laser (0.6 W cm^−2^, 10 min) and then transferred for XPS measurement. High‐resolution Cu 2p spectra were collected and deconvoluted to determine the relative percentages of Cu^+^ and Cu^2+^ species.

In addition, the generation of Cu^+^ was further confirmed using a neocuproine‑based colorimetric method. In a typical procedure, 1 mL of Te@PDA‑Cu^II^ dispersion (200 µg mL^−1^) was mixed with 4 mL of neocuproine solution (0.2 mM in ethanol). The mixture was divided into two aliquots: one was irradiated with a NIR‐II laser (0.6 W cm^−2^, 10 min), while the other was kept in the dark as a control. After irradiation, the mixtures were centrifuged (10 000 rpm, 5 min), and the supernatants were analyzed by UV‑‐vis spectroscopy. The characteristic absorption peak at 452 nm, corresponding to the [Cu(neocuproine)_2_]^+^ complex, was used to confirm the presence of Cu^+^.

### 5.7 Cellular Experiments

The mouse mammary carcinoma cell line 4T1 (RRID: CVCL_0125), the human breast adenocarcinoma cell line MCF‐7 (RRID: CVCL_B5PF), and the mouse embryonic fibroblast cell line NIH‐3T3 (RRID: CVCL_0594) were sourced from the Shanghai Institute of Cell Biology, Chinese Academy of Sciences. Cells were cultured in RPMI 1640 medium supplemented with 10% fetal bovine serum (FBS) and 1% penicillin‐streptomycin at 37°C in a 5% CO_2_ humidified incubator. Prior to experimentation, 4T1 cells underwent authentication and were confirmed to be free of mycoplasma contamination.

### 5.8 Cellular Uptake and Intracellular Copper Ion Detection

To detect cellular uptake of Te@PDA‐Cu^II^, 4T1 cells were seeded at a density of 1 × 10^5^ cells per well in a 12‐well plate. After 24 h of incubation, DiD‐labeled Te@PDA‑Cu^II^ (200 µg mL^−1^) were added and further incubated for 1, 2, and 4 h, respectively. Cells were then washed with PBS and fixed. Cellular uptake was visualized using CLSM.

Then, to detect the intracellular copper ion contents, 4T1 cells were seeded at a density of 1 × 10^5^ cells per well in a 12‐well plate and incubated for 24 h. The cells were then treated with Te@PDA‑Cu^II^ (200 µg mL^−1^) for 1, 2, or 4 h, respectively. After treatment, the cells were incubated with 10 µM copper ion‑sensitive fluorescent probe RBH working solution for 20 min. Subsequently, intracellular copper ion levels were analyzed using both CLSM and flow cytometry.

### 5.9 Detection of Intracellular ROS Generation

Intracellular ROS levels were assessed using the fluorescent probe DCFH‑DA. Briefly, 4T1 cells were seeded in 12‑well plates (1 × 10^5^ cells/well) and cultured for 24 h, followed by treatment with PBS, Te@PDA, or Te@PDA‑Cu^II^ at an equivalent concentration of 200 µg mL^−1^ for 4 h with or without irradiated by a 1064 nm NIR‑II laser (0.6 W cm^−2^, 10 min). After continuing to incubate for 2 h, all groups were incubated with 10 µM DCFH‑DA at 37 °C for 30 min, washed with PBS, and analyzed. Intracellular ROS fluorescence was quantified by flow cytometry and visualized using confocal laser scanning microscopy.

### 5.10 Detection of Mitochondrial Damage

Mitochondrial damage was assessed by detecting changes in mitochondrial membrane potential using the JC‐1 fluorescent probe. Briefly, 4T1 cells seeded in 12‐well plates (1 × 10^5^ cells/well) were treated after 24 h of culture with PBS, Te@PDA, or Te@PDA‐Cu^II^ at an equivalent concentration of 200 µg mL^−1^ for 4 h and irradiated with or without a 1064 nm NIR‐II laser (0.6 W cm^−2^, 10 min). After 24 h, cells were incubated with JC‐1 staining solution (5 µg/mL) at 37°C for 0.5 h, washed with PBS, and analyzed. The red‐to‐green fluorescence intensity ratio was quantified using flow cytometry.

### 5.11 Detection of Apoptosis

Apoptosis was evaluated using an Annexin V‐FITC/PI detection kit. 4T1 cells were seeded in 6‐well plates (2 × 10^5^ cells/well) and cultured for 24 h, followed by the same treatment and irradiation conditions described above. After treatment, cells were harvested, washed with PBS, and resuspended in 100 µL of 1× Binding Buffer. Then, 5 µL of Annexin V‐FITC and 5 µL of PI were added, and the cells were incubated at room temperature in the dark for 15 min. Apoptotic cell populations were quantified by flow cytometry.

### 5.12 Detection of Cell Viability

Cell viability was assessed using the MTT assay. In general, 4T1 cells were seeded in 96‐well plates at a density of 1 × 10^4^ cells per well and cultured for 24 h. For the concentration‐dependent experiment, cells were treated with various concentrations of Te@PDA‐Cu^II^ (0, 12.5, 25, 50, 100, and 200 µg mL^−1^) for 4 h, with or without a 1064 nm NIR‐II laser irradiation (0.6 W cm^−2^, 10 min). 24 h later, the cell viability was detected by MTT assay using a microplate reader. The same procedure was also performed on NIH‐3T3 cells under the same conditions to evaluate the biocompatibility of the nanoreactor. In addition, cells were treated with PBS, Te@PDA, and Te@PDA‐Cu^II^ at an equivalent concentration of 200 µg mL^−1^, followed by being exposed to 1064 nm NIR‐II laser irradiation (0.6 W cm^−2^, 10 min). The MTT procedure was performed as described above.

### 5.13 Detection of Cuproptosis

4T1 cells were seeded on glass coverslips in 12‐well plates at a density of 1 × 10^5^ cells per well and cultured for 24 h. Cells were then treated with PBS, Te@PDA, or Te@PDA‐Cu^II^ at an equivalent concentration of 200 µg mL^−1^ for 4 h, followed by irradiation with a 1064 nm NIR‐II laser (0.6 W cm^−2^, 10 min). After 12 h of treatment, cells were incubated with Mito‐Tracker Red for 30 min to label mitochondria. Subsequently, cells were fixed with 4% paraformaldehyde for 15 min, permeabilized with 0.1% Triton X‐100 for 10 min, and blocked with 5% BSA for 1 h at room temperature. Cells were then incubated overnight at 4°C with primary antibodies against DLAT (1:1000), FDX1 (1:1000), and LIAS (1:1000), respectively. After washing, cells were incubated with corresponding fluorescent secondary antibodies for 1 h at room temperature in the dark. Nuclei were counterstained with DAPI. Fluorescence images were captured using a CLSM, and mean fluorescence intensity was quantified using ImageJ software. In parallel, MCF‐7 cells were subjected to the identical treatment protocol to assess the expression changes of DLAT, FDX1, and LIAS following the same treatments.

Besides, with the same cell treatments, the expression levels of DLAT, FDX1, and LIAS were assessed by Western Blot analysis

### 5.14 Detection of Immunogenic Cell Death

4T1 cells were seeded on glass coverslips in 12‑well plates at a density of 1 × 10^5^ cells per well and cultured for 24 h. After treatment with PBS, Te@PDA, or Te@PDA‑Cu^II^ (200 µg mL^−1^, 4 h), with or without subsequent NIR‑II laser irradiation (0.6 W cm^−2^, 10 min). After 24 h, cells were washed with PBS and fixed with 4% paraformaldehyde for 15 min without permeabilization to specifically detect surface‑exposed CRT. For HMGB1 release, cells were fixed and permeabilized with 0.1% Triton X‑100. After blocking with 5% BSA for 1 h, cells were incubated overnight at 4°C with primary antibodies against CRT (1:1000) or HMGB1 (1:1000), followed by incubation with fluorescent secondary antibodies (1:1000) for 1 h at room temperature in the dark. Nuclei were counterstained with DAPI. Fluorescence images were captured using a CLSM, and the percentage of CRT‑positive cells or HMGB1 nuclear‑to‑cytoplasmic translocation was quantified using ImageJ. In addition, cell culture supernatants were collected for extracellular ATP detection using a commercial ATP assay kit. Meanwhile, MCF‐7 cells were subjected to the same treatment protocol to evaluate CRT surface exposure, HMGB1 release, and ATP secretion following NIR‐II laser irradiation.

### 5.15 Assessment of Dendritic Cell Maturation and Macrophage Polarization

To detect dendritic cell maturation induced by Te@PDA‐Cu^II^ thermoelectric nanoreactors. Bone marrow‑derived dendritic cells (BMDCs) were isolated from the femurs and tibiae of BALB/c mice and cultured in RPMI‑1640 medium supplemented with 20 ng mL^−1^ GM‑CSF for 7 days. Immature BMDCs were then co‑cultured with conditioned medium from 4T1 cells treated under different experimental conditions (PBS, Te@PDA, Te@PDA‑Cu^II^, with or without NIR‑II laser irradiation) for 24 h. Cells were harvested, washed with PBS, and stained with fluorochrome‑conjugated antibodies against CD11c, CD80, CD86, and CD40 for 1 h at 4°C in the dark. After washing, the percentage of CD11c^+^ cells expressing high levels of CD80, CD86, and CD40 was analyzed using flow cytometry.

To assess the potential of macrophage polarization, mouse bone marrow‐derived macrophages (BMDMs) were seeded in 12‑well plates at a density of 2 x 10^5^ cells per well and allowed to adhere overnight. Cells were then treated with conditioned medium from differently treated 4T1 cells (as described above) for 24 h. Cells were harvested, washed with PBS, and stained with fluorochrome‑conjugated antibodies against F4/80, CD80, and CD206 for 1 h at 4°C in the dark, then analyzed using flow cytometry.

### 5.16 Animal Experiments

Female BALB/c mice (6–8 weeks old) were purchased from Jinan Xingkang Biotechnology Co., Ltd. All animal procedures were performed in accordance with the institutional guidelines and were approved by the Animal Welfare and Research Ethics Committee of Shandong First Medical University (Approval No. W202510270922) and maintained under specific pathogen‐free (SPF) conditions. To establish the orthotopic breast tumor model, luciferase‐expressing 4T1 (4T1‐Luci) cells were harvested and resuspended in PBS. ∼2 × 10^6^ cells in 50 µL were injected into the mammary fat pad of each mouse under anesthesia. For the subcutaneous model, the same number of 4T1 cells was inoculated into the right flank of each mouse.

### 5.17 In Vivo Antitumor Effect of Te@PDA‐Cu^II^ Thermoelectric Nanoreactors

Female BALB/c mice bearing subcutaneous 4T1 breast tumors were used. When tumor volumes reached ∼60 mm^3^, mice were randomly divided into five groups (n  =  5 per group): (1) PBS, (2) PBS ^+^ NIR‑II, (3) Te@PDA + NIR‑II, (4) Te@PDA‑Cu^II^, (5) Te@PDA‑Cu^II^ + NIR‑II. Mice in the designated groups received an intratumoral injection of the corresponding reagents at an equivalent concentration of 1 mg kg^−1^. After 4 h, tumors in the irradiated groups were exposed to a 1064 nm NIR‑II laser at a power density of 0.6 W cm^−2^ for 5 min. During irradiation, real‑time thermal images and temperature data of the tumor region were recorded using an infrared thermal imaging camera. For tumor size monitoring, the longest diameter and width were measured every other day, with volume calculated as: V = width^2^ × length / 2. Also, the weights of mice were recorded every other day. On day 1 post‐treatment, tumor tissues were harvested and processed for histological and immunofluorescence analysis. Sections were stained with hematoxylin and eosin (H&E) for morphology analysis, while immunofluorescence staining was performed to assess the expression of CRT, HMGB1, and cuproptosis‐related markers (e.g., FDX1, DLAT, LIAS).

For the orthotopic breast cancer model, bioluminescence imaging was performed using an IVIS Spectrum CT system after intraperitoneal injection of luciferase to monitor the growth dynamics of 4T1‐Luci tumors every four days. In addition, the tumor sizes were recorded by vernier caliper. Following a 12‐day treatment, mice were sacrificed for analysis of lung metastasis. Lungs were carefully excised and immediately subjected to ex vivo bioluminescence imaging using the IVIS Spectrum CT system to detect the presence and distribution of metastatic 4T1‐Luci nodules. Subsequently, the lungs were fixed in 4% paraformaldehyde, embedded in paraffin, and sectioned. Hematoxylin and eosin (H&E) staining was performed on the tissue sections to visualize metastatic foci and assess the extent of lung colonization.

### 5.18 Immunological Evaluation of Te@PDA‐Cu^II^ Thermoelectric Nanoreactors

To elucidate the immunological mechanisms underlying the antitumor efficacy of the Te@PDA‐Cu^II^ thermoelectric nanoreactors, mice were treated as above. On day 5 post various treatments, tumors were harvested and processed into single‐cell suspensions. DC maturation was evaluated by flow cytometry based on the co‐expression of CD80 and CD86. Tumor‐infiltrating lymphocytes were analyzed for the proportions of CD3^+^CD8^+^ cytotoxic T cells, and their functional states were determined by intracellular staining for Granzyme B and IFN‐γ. In parallel, the concentrations of key antitumor cytokines (TNF‐α, IFN‐γ, IL‐6) in both tumor homogenates and serum were quantified using enzyme‐linked immunosorbent assay (ELISA) kits.

### 5.19 Abscopal Effect of Te@PDA‐Cu^II^ Thermoelectric Nanoreactors in Combination with aPD‑1 Therapy

To evaluate the systemic antitumor immune response induced by the thermoelectric nanoreactor Te@PDA‐Cu^II^ in combination with aPD‐1 checkpoint blockade, a bilateral subcutaneous 4T1 tumor model was established in female BALB/c mice. On day ‐7, 2 × 10^6^ 4T1 cells in 50 µL PBS were injected into the right flank to form the primary tumor. On day ‐4, an identical inoculum was injected into the left flank to establish the secondary tumor, mimicking a metastatic focus. When the primary tumors reached ∼40 mm^3^, mice were randomly allocated into four groups (n  =  5 per group): (1) Control, (2) Te@PDA‐Cu^II^ + NIR‐II, (3) aPD‐1 antibody, (4) Te@PDA‐Cu^II^ + NIR‐II + aPD‐1 antibody. On day 0, primary tumors in groups 2 and 4 received an intratumoral injection of Te@PDA‑Cu^II^ at a dose of 1 mg kg^−1^. 4 h post‑injection, these tumors were exposed to 1064 nm NIR‑II laser irradiation (0.6 W cm^−2^ for 5 min). Mice in groups 3 and 4 were administered aPD‑1 antibody via tail vein injection at a dose of 20 µg per mouse on days 2, 4, and 6. Both primary and distant tumors were monitored throughout the treatment period.

### 5.20 Long‐Term Immunological Memory Effect of Te@PDA‐Cu^II^ Thermoelectric Nanoreactors in Combination with aPD‑1 Therapy

To evaluate the establishment of long‐term antitumor immunological memory, a unilateral subcutaneous 4T1‐Luci tumor model was established in female BALB/c mice. When tumor volumes reached ∼60 mm^3^, mice were randomly divided into four treatment groups as previously described. Primary tumors were treated locally, and systemic aPD‐1 therapy was administered according to the established protocol. Mice that achieved complete tumor regression and remained tumor‐free for 60 days were designated as “cured”. On day 60 post‐treatment, blood samples were collected via retro‐orbital bleeding, and the frequencies of central memory T cells (Tcm, CD8^+^CD44^+^CD62L^+^) and effector memory T cells (Tem, CD8^+^CD44^+^CD62L^−^) were analyzed by flow cytometry. Simultaneously, serum levels of the cytokines TNF‐α and IFN‐γ were quantified using ELISA kits.

To directly assess protective immunity, on day 65 post‐cure, the cured mice were rechallenged by subcutaneous injection of 2 × 10^6^ 4T1‐Luci cells into the mammary fat pad. Age‐matched naive BALB/c mice receiving the same tumor inoculum served as the control group. Tumor growth was monitored every two days. At day 75, all mice were euthanized, and their lungs were harvested for analysis of metastasis.

### 5.21 Statistical Analysis

The experimental data presented in this work were obtained from at least three independent replicates. All statistical evaluations were carried out with GraphPad Prism 9.0, employing one‐way ANOVA for intergroup comparisons. Data are reported as mean ± SEM. Statistical significance is denoted as follows: ^*^ *p* < 0.05, ^**^ *p* < 0.01, ^***^ *p* < 0.001.

## Author Contributions

**Shining Yang**: data curation, investigation. **Yuechao Yang**: software, methodology. **Xin Wang**: funding acquisition, visualization, writing – review and editing. **Shushu Chu**: methodology, software, data curation. **Xinran Qu**: data curation, methodology. **Jinqiao Zhang**: methodology, investigation. **Boyu Yuan**: methodology, data curation, project administration, validation, investigation, writing – original draft, visualization. **Ziliang Dong**: writing – review and editing, supervision, resources, funding acquisition, conceptualization. **Yiju Wei**: visualization, formal analysis. **Qin Fan**: conceptualization, visualization, writing – original draft. **Chenxi Zhang**: methodology, investigation.

## Conflicts of Interest

The authors declare no conflicts of interest.

## Supporting information




**Supporting File**: advs76368‐sup‐0001‐SuppMat.docx.

## Data Availability

The data that support the findings of this study are available from the corresponding author upon reasonable request.
