# Supplementary material for: NIR‐II Light‐Triggered Electron Flow Initiates Cuproptosis‐Centered Thermoelectric‐Immunotherapy for Breast Cancer
Source: Adv Sci (Weinh). 2026 Jun 30;13(53):e76368. doi: 10.1002/advs.76368 (PMC13336946; doi:10.1002/advs.76368)
Supplement: Supplementary file 1 [file ADVS-13-e76368-s001.docx]

**NIR-II Light-Initiated Electron Flow Ignites Cuproptosis-Centered Thermoelectric-Immunological Cascade for Breast Cancer Treatment**

Boyu Yuan^1,2^, Qin Fan^4^*, Shushu Chu^2^, Shining Yang^2^, Yuechao Yang^2^, Chenxi Zhang^3^, Jinqiao Zhang^2^, Xinran Qu^2^, Yiju Wei^5^, Xin Wang^3^*, Ziliang Dong^1,2^*

1. Department of Clinical Pharmacy, The First Affiliated Hospital of Shandong First Medical University & Shandong Provincial Qianfoshan Hospital, Shandong Key Laboratory of Digital Diagnosis and Treatment of Thoracic Oncology, Jinan, Shandong, 250014, P. R. China.
2. Science and Technology Innovation Center, Shandong First Medical University & Shandong Academy of Medicine Sciences, Jinan, Shandong, 250117, P. R. China
3. Faculty of Light Industry, State Key Laboratory of Green Papermaking and Resource Recycling, Qilu University of Technology (Shandong Academy of Sciences), Jinan 250353, P. R. China
4. State Key Laboratory of Flexible Electronics (LoFE) & Institute of Advanced Materials (IAM), Nanjing University of Posts & Telecommunications, Nanjing, Jiangsu, 210000, P. R. China
5. School of Life Science, Shandong First Medical University & Shandong Academy of Medica science, Tai’an, 271016, Shandong, China

E-mail: [wangxinpolymer@qlu.edu.cn](mailto:wangxinpolymer@qlu.edu.cn), [iamqfan@njupt.edu.cn](mailto:iamqfan@njupt.edu.cn), [dongziliang@sdfmu.edu.cn](mailto:dongziliang@sdfmu.edu.cn)

**Figure S1**. Zeta potentials of Te NRs, Te@PDA, and Te@PDA-Cu^II^.

**Figure S2**. FTIR spectra of Te NRs, Te@PDA and Te@PDA-Cu^II^.

**Figure S3**. Photothermal curves of Te@PDA-Cu^II^ (200 μg mL^−1^) irradiated by a NIR-II laser at diverse power densities.

**Figure S4**. Photothermal curve of Te@PDA-Cu^II^ (100 μg mL^-1^) under alternating NIR-II laser exposure (0.6 W cm^-2^, 5 min/cycle).

**Figure S5**. Energy bandgap of Te NRs and Te@PDA.

**Figure S6**. (A-C) Electron Localization Function (ELF) maps of the DHI monomer A), DHI dimer B), and DHI trimer C).

**Figure S7**. High-resolution Cu 2p XPS spectra of the Te@PDA-Cu^II^ nanorods before A) and after B) 1064 nm NIR-II laser irradiation.

**Figure S8**. Time-dependent intracellular copper ion accumulation detected by flow cytometry.

**Figure S9.** Relative cell viability of 4T1 cells under different treatments, as indicated.

**Figure S10.** Relative viability of NIH-3T3 cells treated with various concentrations of Te@PDA-Cu^II^ with or without NIR-II laser irradiation.

**Figure S11**. Immunofluorescence staining of LIAS and corresponding quantification of average fluorescence intensity under different treatments.

**Figure S12.** Relative viability of 4T1 cells following different treatments, as indicated.

**Figure S13.** (A-C) Immunofluorescence images of DLAT A), FDX1 B) and LIAS C) in MCF-7 cells following different treatments. (D-F) Corresponding quantitative mean fluorescence intensity (MFI) of DLAT D) FDX1 E) and LIAS F) in MCF-7 cells.

**Figure S14**. Time-dependent UV-Vis-NIR absorption spectra of DPBF in the presence of Te@PDA-Cu^II^ under NIR-II laser irradiation.

**Figure S15**. Quantitative analysis of changes in mitochondrial membrane potential in 4T1 cells following different treatments.

**Figure S16.** (A, B) Immunofluorescence images A) and corresponding quantitative mean fluorescence intensity (MFI) B) of CRT in MCF-7 cells following different treatments. (C, D) Immunofluorescence images C) and corresponding MFI D) of HMGB1 following different treatments. (E) Detection of extracellular ATP secretion levels in MCF-7 cells after different treatments.

**Figure S17**. Hemolysis rate spectrum of blood incubated with Te@PDA-Cu^II^ at different concentrations. Negative control: whole blood in 1 × PBS. Positive control: whole blood in deionized water.

**Figure S18.** (A-C) Corresponding quantitative analysis of the mean fluorescence intensity (MFI) of the immunofluorescence assays for DLAT A), FDX1 B), and LIAS C).

**Figure S19**. Immunofluorescence staining and corresponding quantitative analysis of HMGB1 and CRT proteins in tumor tissues.

**Figure S20**. Flow cytometric plots and quantitative analysis of M1 macrophages (F4/80^+^CD80^+^) in tumor tissues.

**Figure S21**. (A-B) Flow cytometry profiling and corresponding quantitative analysis of Interferon-γ A) and Granzyme B B) in tumor-infiltrating CD8⁺ T cells.

**Figure S22**. Survival curves of 4T1-Luci tumor-bearing mice after different treatments.

**Figure S23**. Individual tumor growth curves of cured mice after rechallenge with 4T1-Luci cells.

**Figure S24**. Quantification of lung metastatic nodules on day 10 post-rechallenge.

**Figure S25.** Blood biochemistry and hematology analysis of healthy mice and mice injected with Te@PDA-Cu^II^. Data were presented as mean ± SEM.
